# Preventive Medications in Pediatric Migraine: A Network Meta-Analysis

**DOI:** 10.1001/jamanetworkopen.2024.38666

**Published:** 2024-10-10

**Authors:** Omid Kohandel Gargari, Sepehr Aghajanian, Mansoureh Togha, Fateme Mohammadifard, Romina Abyaneh, Sheida Mobader Sani, Reza Samiee, Ali Kermanpour, Niloofar Seighali, Faraidoon Haghdoost

**Affiliations:** 1 Headache Department, Iranian Center of Neurological Research, Neuroscience Institute, Tehran University of Medical Sciences, Tehran, Iran; 2 School of Medicine, Alborz University of Medical Sciences, Karaj, Iran; 3 Neuroscience Research Center, Iran University of Medical Sciences, Tehran, Iran; 4 Headache Department, Neurology Ward, Sina University Hospital, School of Medicine, Tehran University of Medical Sciences, Tehran, Iran; 5 NeuroWeb Association, Students’ Scientific Research Center (SSRC), Tehran University of Medical Sciences, Tehran, Iran; 6 The George Institute for Global Health, University of New South Wales, Sydney, Australia

## Abstract

**Importance:**

Pediatric migraine substantially impacts quality of life and academic performance among children and adolescents. Understanding the efficacy and safety of pharmacological interventions for migraine prophylaxis in this population is crucial for developing effective treatment strategies.

**Objective:**

To conduct a comprehensive network meta-analysis to evaluate the efficacy and safety associated with pharmacological treatments for pediatric migraine prophylaxis among pediatric patients with a migraine diagnosis and assess interventions involving various oral pharmacological interventions compared with each other and placebo.

**Data Sources:**

PubMed, Embase, and SCOPUS were searched for publications up to September 2023. Search terms and indexing were chosen to encompass relevant studies, focusing on randomized clinical trials in pediatric migraine prophylaxis.

**Study Selection:**

Inclusion criteria targeted randomized clinical trials involving pediatric patients with migraine. Studies were selected based on their examination of oral pharmacological interventions. The search yielded an initial 9162 citations.

**Data Extraction and Synthesis:**

Data extraction adhered to Preferred Reporting Items for Systematic Reviews and Meta-Analyses reporting guidelines. Five investigators independently extracted study data into a spreadsheet in duplicate. Study-level estimates were calculated, employing a random-effects model for primary and secondary outcomes due to identified heterogeneity. Data analysis was conducted from December 2023 to March 2024

**Main Outcomes and Measures:**

The primary outcome was migraine frequency (number of attacks per month). Secondary outcomes included a 50% or greater responder rate, headache duration, headache intensity, and disability (assessed by pediatrics migraine-specific disability tool). Adverse events were also evaluated.

**Results:**

The analysis incorporated 45 trials with 3771 participants. Compared with placebo, pregabalin (ratio of means [RoM], 0.38; 95% CI, 0.18-0.79) and topiramate with vitamin D3 (RoM, 0.44; 95% CI, 0.30-0.65) were associated with reduction in migraine frequency. Flunarizine (RoM, 0.46; 95% CI, 0.26-0.81), levetiracetam (RoM, 0.47; 95% CI, 0.30-0.72), riboflavin (RoM, 0.50; 95% CI, 0.32-0.77), cinnarizine (RoM, 0.64; 95% CI, 0.46-0.88), topiramate (RoM, 0.70; 95% CI, 0.55-0.89), and amitriptyline (RoM, 0.73; 95% CI, 0.54-0.97) were also associated with reduction in migraine frequency, but these findings were drawn from individual studies. For the 50% or greater responder rate, flunarizine and α-lipoic acid (risk ratio [RR], 8.73; 95% CI, 2.44-31.20), flunarizine (RR, 4.00; 95% CI, 1.38-11.55), pregabalin (RR, 1.88; 95% CI, 1.13-3.14), and cinnarizine (RR, 1.46; 95% CI, 1.04-2.05) were associated with significantly greater effectiveness than placebo. Compared with placebo, propranolol and cinnarizine (RoM, 0.45; 95% CI, 0.28-0.72), pregabalin (RoM, 0.57; 95% CI, 0.33-0.96), valproate (RoM, 0.60; 95% CI, 0.49-0.72), levetiracetam (RoM, 0.62; 95% CI, 0.50-0.77), and cinnarizine (RoM, 0.64; 95% CI, 0.54-0.76) were significantly associated with reduction in headache intensity. However, no treatments were associated with significant improvements in quality of life or reduction of the duration of migraine attacks. Adverse events were higher with amitriptyline (RR, 3.81; 95% CI, 1.41-10.32), topiramate (RR, 4.34; 95% CI, 1.60-11.75), and valproate (RR, 5.93; 95% CI, 1.93-18.23) compared with placebo.

**Conclusions and Relevance:**

In this network meta-analysis of randomized clinical trials, topiramate and pregabalin were associated with reduction in headache frequency and intensity. Although there were also other drugs that showed statistically significant results (flunarizine, riboflavin, amitriptyline, and cinnarizine), more studies were required for a robust conclusion. None of the drugs were associated with improved quality of life or attack duration, underscoring the need for further research to develop more comprehensive treatment strategies and explore the potential of combination therapies, especially those involving vitamins. Future studies should focus on validating these findings and expanding the treatment landscape for pediatric migraine management.

## Key Points

**Question:**

What is the efficacy, safety, and acceptability associated with pharmacological treatments for pediatric migraine prophylaxis?

**Findings:**

This network meta-analysis of 45 clinical trials showed that pregabalin, topiramate (with and without vitamin D3), flunarizine, levetiracetam, riboflavin, and cinnarizine were associated with a reduction in migraine frequency compared with placebo. Pregabalin, flunarizine, α-lipoic acid, and cinnarizine had a significantly higher 50% reduction rate, and propranolol and cinnarizine, pregabalin, valproate, and levetiracetam were associated with a reduction in pain intensity compared with placebo.

**Meaning:**

These findings suggest that pharmacological treatments like pregabalin and topiramate, especially with vitamin supplements, may reduce migraine frequency and intensity in pediatric patients.

## Teaser

This meta-analysis evaluates the efficacy and safety associated with various pharmacological treatments for pediatric migraine.

## Introduction

Primary headaches, particularly migraine, are common neurological disorders during childhood and adolescence. The estimated overall prevalence of migraine in children and adolescents is 11%.^1^ Migraine incidence rises notably among individuals aged 14 and older, showing a sex predilection for females in adolescence and young adulthood, as opposed to younger age groups.^2^ Migraine has increasingly become a leading cause of disability, and headache episodes have a substantial impact on quality of life and academic performance.^3^ Furthermore, it is suggested to increase the likelihood of developing psychological disorders including depression and anxiety disorders.^4^

The *International Classification of Headache Disorder* (*ICHD*) was introduced by the International Headache Society in 1988. The latest edition, *ICHD-III*, was published in 2018, providing an essential tool based on headache characteristics including duration, location, quality of headaches, and accompanying symptoms, ultimately facilitating precise diagnosis and effective treatment.^5^ Despite migraine’s relatively straightforward diagnosis based on the *ICHD* criteria, treatment has emerged as a growing concern. Migraine treatment typically comprises 2 principal strategies: acute or abortive medications for immediate relief and prophylactic treatment to prevent future migraine attacks and to stop the condition from becoming chronic. First-line medication used for acute management of migraine in younger children consists of acetaminophen, ibuprofen, and other nonsteroidal anti-inflammatory drugs. Triptans are proven effective for treating moderate to severe headaches in adolescents. Combinational therapies, like sumatriptan and naproxen, have shown promise in managing adolescent migraine.^6,7,8,9^ Rizatriptan exclusively holds US Food and Drug Administration (FDA) approval for children older than 6 years, whereas sumatriptan, zolmitriptan, and almotriptan have received FDA endorsement for adolescents aged 12 years and older.^10,11^

Prophylactic treatment of pediatric migraines presents a challenging landscape due to limited evidence. Off-label use of preventive medication is common, highlighting the need for additional research and guidelines. Although topiramate is the first FDA-approved preventive medication for adolescent migraine, studies show less favorable results compared with a placebo, which contrasts with findings in adults. Evaluating treatment efficacy in pediatric and adolescent patients with migraine is further complicated by a significant placebo effect.^7,12^ This network meta-analysis (NMA) aims to conduct an in-depth examination of the existing evidence regarding the efficacy, safety, and acceptability of pharmacological treatments for pediatric migraine prophylaxis.

## Methods

This systematic review and meta-analysis incorporated randomized clinical trials investigating the pharmacological interventions as delineated in the review protocol registered in PROSPERO (CRD42024496262). The reporting of the study adhered to the Preferred Reporting Items for Systematic Reviews and Meta-Analyses (PRISMA) reporting guideline.^13,14,15^

### Search Strategy and Study Selection

PubMed, Embase, SCOPUS, and ClinicalTrials.gov were searched for eligible studies from inspection to September 2023. The search query is provided in eAppendix in Supplement 1. Citations were also extracted from a previous similar NMA.^16^ After the initial search, 5 investigators (S.M.S., R.S., R.A., A.K., and N.S.) screened titles and abstracts independently in duplicate. The next step was full-text screening, completed by 3 investigators (O.K.G., S.M.S., and R.S.) who screened full-text of selected studies in duplicate and assessed their eligibility based on the following population, intervention, comparison, outcomes, and study type (PICOT) criteria. The population (ie, target demographic) included individuals younger than 18 years, encompassing children and adolescents. Prospective participants must have had a confirmed diagnosis of migraine, with or without aura, as per the criteria established by the *ICHD-III*.^17^ Alternatively, their migraine diagnosis must have aligned closely with the classification outlined by the International Headache Society. The intervention was oral pharmacological drugs. Comparisons included head-to head comparison of 2 oral pharmacological drugs with each other or placebo. Outcomes included headache frequency (number of migraine attacks per month), 50% or greater responder rate (50% headache frequency reduction rate), headache duration (mean duration of migraine attacks), headache intensity (self-reported pain score), and disability produced by migraine assessed by pediatrics migraine-specific disability tool (PedMIDAS).^18^ Study type included randomized clinical trials.

### Data Extraction

Five coauthors (O.K.G., R.A., S.M.S., R.S., and A.K.) divided included studies and extracted characteristics and outcomes into an Excel sheet in duplicate. Disagreements were resolved through group discussion.

### Outcomes

Our primary outcome was absolute headache frequency after treatment, defined as number migraine attacks per month. For studies with multiple follow-up times, the last follow-up time was selected for analysis.

Secondary outcomes included 50% or greater responder rate, defined as number of patients with at least 50% reduction in headache frequency after treatment compared with baseline, and headache duration, which is defined as duration of each individual attack in hours. Another outcome was disability produced by migraine as measured by the PedMIDAS tool, which is a 6-question tool developed to assess the disability caused by migraines in school-age children and adolescents. It was validated by administering it to 441 patients attending a pediatric headache center.^18^ The final outcome was self-reported headache intensity, which is a numeric assessment of pain intensity from 0 to 10. Just like the primary outcome for studies with more than 1 end point, the last reported end point was selected. Additionally, the overall number of adverse events was monitored to assess safety.

### Quality Assessment and Risk of Bias

Quality of included studies was assessed using Cochrane Risk of Bias Tool version 2.^19^ Each study quality was assessed by one author.

### Statistical Analysis

Study level estimates were calculated as ratio of means (RoM) or risk ratio (RR) and 95% CI using the Metafor package in R version 4.3 (R Project for Statistical Computing). Primary and secondary outcomes were each evaluated via the frequentist NMAs based on the graph-theoretical approach using Netmeta version 2.8-2 in R. For all estimates, the reference group was placebo.

Treatment effectiveness and superiority for each outcome were assessed using P-scores, which are analogous to surface under the cumulative ranking curve scores in frequentist statistics. Heterogeneity was quantified through within-design and between-design *Q* statistics, and *I^2^*. The transitivity assumption was investigated by comparing key study characteristics and assessing local (net splitting of direct and indirect evidence for all available comparisons) and global consistency (design-by-treatment interaction test) of inconsistency.

Utilizing the data from the common components in treatment groups of the studies, we evaluated the effect of multicomponent treatments using supplements and their potential additive effect to the most efficacious single-agent pharmacological treatment for the primary outcome using component meta-analysis. All parameters were estimated using weighted least squares regression.

To evaluate the effect of baseline study confounders, including migraine diagnosis method, dichotomized mean age of the participants and proportion of male sex in the studies, and inclusion of patients with migraine with aura, a secondary random-effects analysis within the bayesian framework was carried out for the primary outcome. Noninformative priors were selected as implemented in the Gemtc package version 1.0-2 in R. To ensure convergence Gelman-Rubin-Brooks plots and the associated potential scale reduction factor were closely inspected. All meta-regression models were carried out with 4 chains, 5000 burn-in iterations, and 106 Markov Chain Monte Carlo simulation iterations. The estimated coefficients for binary covariates were considered insignificant if the 95% credible intervals (CrIs) encompassed the no effect estimate. A priori levels of significance were set at *P* < .05, and all hypothesis tests were 2-sided. Analysis occurred from December 2023 to March 2024.

## Results

The initial search yielded 9162 citations, which were subject to abstract screening. Subsequently, 100 reports underwent full-text screening, of which 45 clinical trials^20,21,22,23,24,25,26,27,28,29,30,31,32,33,34,35,36,37,38,39,40,41,42,43,44,45,46,47,48,49,50,51,52,53,54,55,56,57,58,59,60,61,62,63,64^ (including 3771 participants) were included in this systematic review and NMA (eFigure 1 in Supplement 1). Forty-one studies were 2-group randomized clinical trials^20,21,22,23,24,25,26,27,28,29,30,31,32,33,34,35,36,37,38,39,40,41,42,43,44,45,46,47,48,49,50,51,52,53,54,55,56,57,58,59,60^ while 4 consisted of 3-group studies.^61,62,63,64^ *ICHD* versions I to III were the primary method of diagnosing migraine in the included population. Characteristics of included studies are reported in eTable 1 in Supplement 1. Iran contributed the highest percentage of participants, accounting for 1587 participants (42.08%). Risk of bias assessment revealed that included studies had low or medium risk of bias as reported in eTable 1 in Supplement 1. Components contributing to the overall risk of bias score are presented in eFigure 2 and eFigure 3 in Supplement 1.^65^

### Primary Outcome

#### Frequency

**Figure 1.**
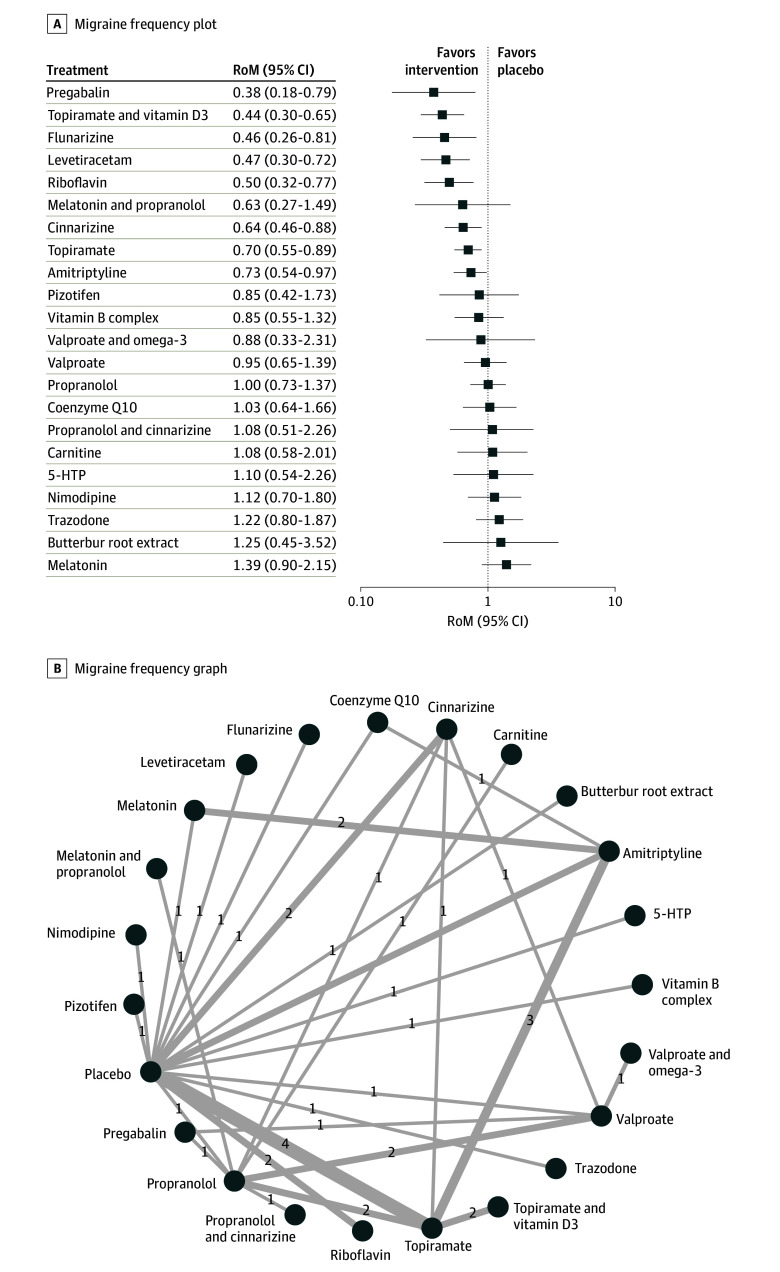
Association of Treatment With Headache Frequency Forest plot (A) and network graph (B) displaying the comparative efficacy of various treatments in reducing the frequency of migraine attacks in pediatric patients. The data includes the ratio of means (RoM) for each treatment compared with placebo, calculated using a random-effects model due to heterogeneity (I^2^ = 67.6%). Significant reductions were observed with pregabalin, topiramate with vitamin d3, flunarizine, levetiracetam, riboflavin, cinnarizine, and amitriptyline. The analysis was based on 37 studies^20,22,23,24,25,26,27,28,29,30,31,32,33,34,35,36,38,39,40,41,42,43,46,47,49,50,51,52,53,54,55,56,57,59,61,63,64^ with 2617 patients. Visual inspection of funnel plots and Begg test confirmed no significant publication bias. 5-HTP indicates 5-hydroxytryptophan.

Of all 45 studies, 37^20,22,23,24,25,26,27,28,29,30,31,32,33,34,35,36,38,39,40,41,42,43,46,47,49,50,51,52,53,54,55,56,57,59,61,63,64^ were eligible for evaluating the efficacy of drugs in reduction of migraine frequency. The analysis was carried out on 2617 patients. The full design-by-treatment interaction *Q* statistic significance (*Q *= 20.86*; P* = .02) and heterogeneity of the analysis (*I^2^* = 67.6%) necessitated the use of a random-effects model. Compared with placebo, pregabalin (RoM, 0.38 95% CI, 0.18-0.79), topiramate with vitamin D3 (RoM, 0.44; 95% CI, 0.30-0.65), flunarizine (RoM, 0.46; 95% CI, 0.26-0.81), levetiracetam (RoM, 0.47 95% CI, 0.30-0.72), riboflavin (RoM, 0.50; 95% CI, 0.32-0.77), cinnarizine (RoM, 0.64; 95% CI, 0.46-0.88), topiramate (RoM, 0.70; 95% CI, 0.55-0.89), and amitriptyline (RoM, 0.73; 95% CI, 0.54-0.97) were treatments significantly associated with lower frequency of migraine. Notably, based on the network estimates, pregabalin resulted in a 62% reduction in migraine frequency. Secondary analysis assuming additive interactions between the treatments extrapolated that the addition of vitamin supplementation with vitamin D3 and riboflavin to pregabalin may elicit a superior response compared with placebo (RoM, 0.12; 95% CI, 0.04-0.30) and pregabalin alone (RoM, 0.31; 95% CI, 0.18-0.54). The top 3 treatments highlighted by P-scores included pregabalin, topiramate and vitamin D3, and levetiracetam (eTable 2 in Supplement 1). The network graph and forest plot for all treatments are visualized in Figure 1. Visual inspection of the funnel plots and Begg test did not reveal any significant publication bias (eFigure 4 in Supplement 1). Details of the pairwise analyses for all available treatments (league table) and network splitting analysis are reported in eTable 3 and eFigure 5 in Supplement 1, respectively. eFigure 6 in Supplement 1 represents the heat map for frequency.

Meta-regression with shared coefficients between study treatments did not identify substantially distinct estimates in studies including patients presenting with aura (RoM, 0.67; 95% CrI, 0.37-1.17) and those using criteria other than *ICHD* for migraine diagnosis (RoM, 0.99; 95% CrI, 0.00-634.06) (eTable 1 in Supplement 1. Furthermore, there was no association with changes in the pooled estimates of the primary outcome among studies with a mean age of the participants greater than 11 years as the mean cut-off (RoM, 1.30; 95% CrI, 0.70-2.33) or male sex dominance (RoM, 0.89; 95% CrI, 0.50-1.58) (eTable 1 in Supplement 1). Additionally, studies with moderate or high risk of bias did not report different estimates compared with other studies evaluated for the primary outcome (RoM, 0.78; 95% CrI, 0.36-1.64) (eTable 1 in Supplement 1).

### Secondary Outcomes

#### 50% or Greater Responder Rate

**Figure 2.**
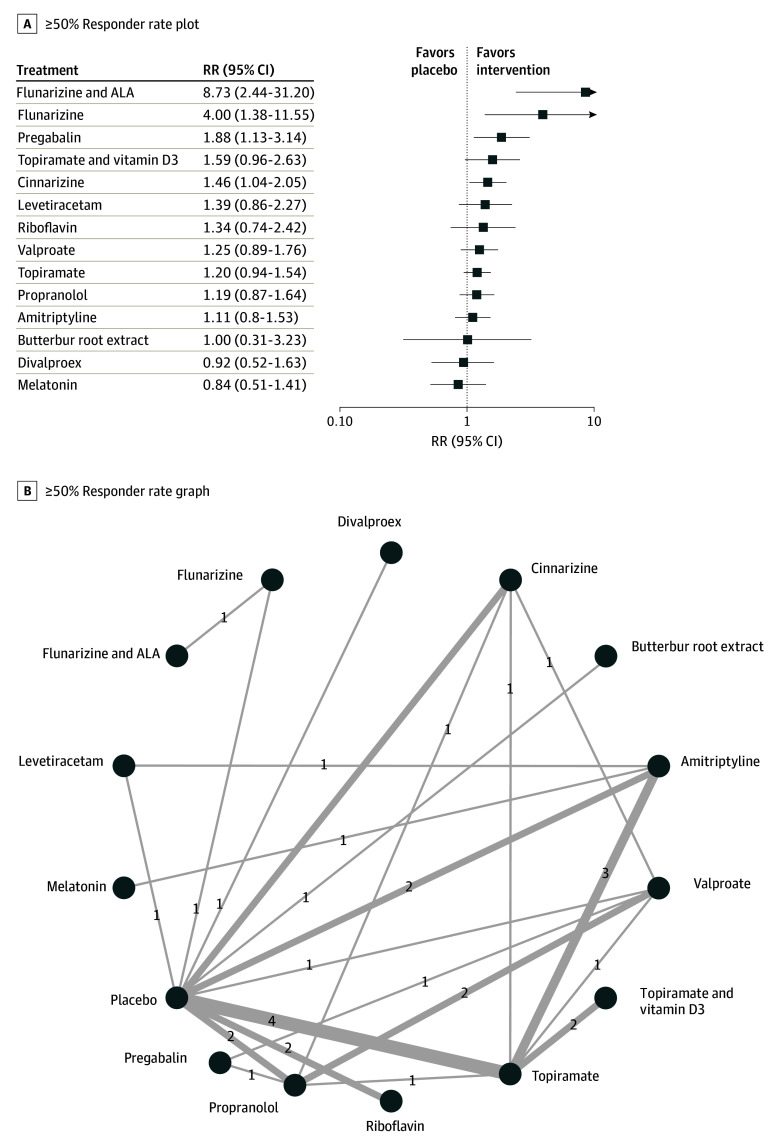
Association of Treatment With 50% or Greater Responder Rate Forest plot (A) and network graph (B) displaying treatments achieving at least a 50% reduction in headache frequency, defined as the number of patients with at least 50% headache frequency reduction after treatment compared with baseline. Risk ratios (RRs) were calculated using a random-effects model due to significant heterogeneity (I^2^ = 70.2%). Flunarizine with α-lipoic acid (ALA), flunarizine alone, pregabalin, and cinnarizine showed significant efficacy compared with placebo. Data were derived from 29 studies^21,22,23,24,25,28,30,31,32,33,37,39,41,42,44,45,46,47,48,51,52,54,55,56,58,60,61,62,64^ involving 2801 patients. No significant publication bias was detected through funnel plot analysis and Begg test.

Of all studies, 29^21,22,23,24,25,28,30,31,32,33,37,39,41,42,44,45,46,47,48,51,52,54,55,56,58,60,61,62,64^ comprising 2801 patients reported a 50% or greater responder rate. Just like the previous outcome, the full design-by-treatment interaction *Q* statistic significance (*Q* = 7.43; P < .001) and heterogeneity of the analysis (*I^2^* = 70.2%) necessitated the use of a random-effects model. Four medications showed a significant effect size compared with placebo, including flunarizine and α-lipoic acid (RR, 8.73; 95% CI, 2.44-31.20), flunarizine (RR, 4.00; 95% CI, 1.38-11.55), pregabalin (RR, 1.88; 95% CI, 1.13-3.14) and cinnarizine (RR, 1.46; 95% CI, 1.04-2.05). Figure 2 presents the forest plot and network graph for all available medications. Visual inspection of the funnel plots and Begg test did not reveal any significant publication bias (eFigure 7 in Supplement 1). Details of pairwise comparison of all available treatment and node splitting analysis results are provided in eTable 4 and eFigure 8 in Supplement 1, respectively. eFigure 9 in Supplement 1 represents the NMA heat map for 50% or greater responder rate.

#### Headache Intensity

**Figure 3.**
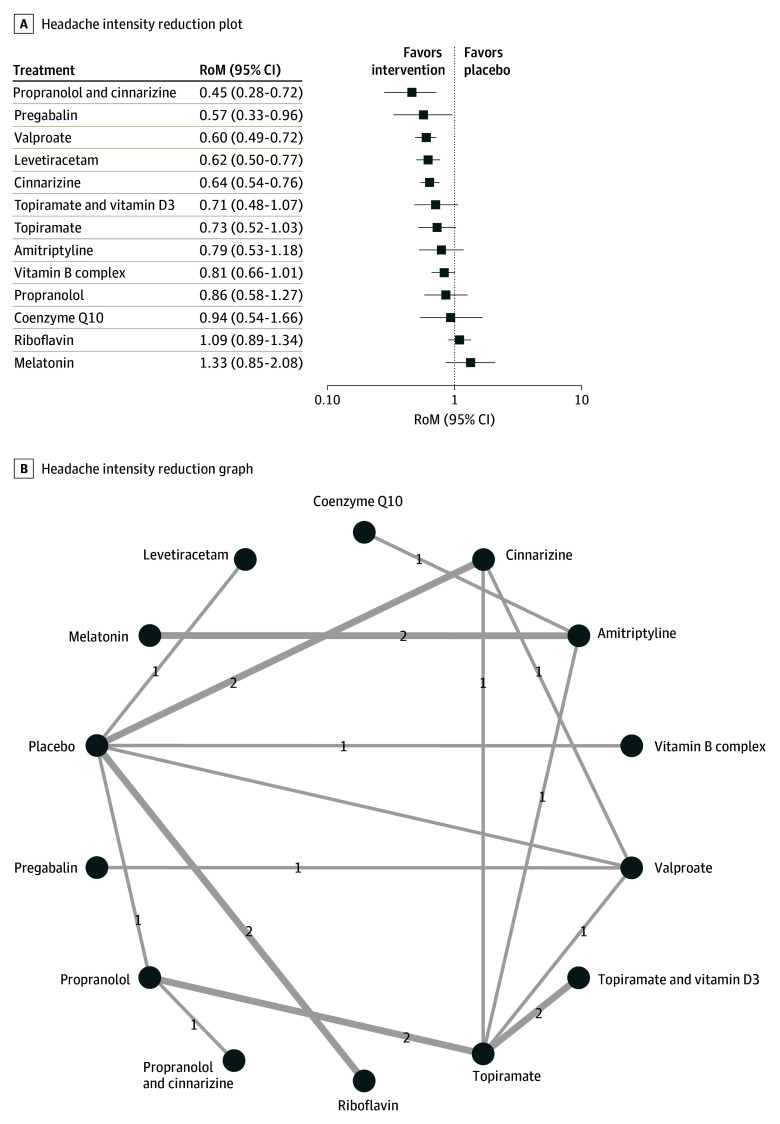
Association of Treatment With Headache Intensity Forest plot (A) and network graph (B) displaying treatments that were associated with a significant reduction of the self-reported intensity of headaches. Ratio of means (RoM) values were calculated using a random-effects model (I^2^ = 43.5%). Effective treatments included propranolol with cinnarizine, pregabalin, valproate, levetiracetam, and cinnarizine. The analysis incorporated 19 studies.^22,23,29,30,31,32,33,39,40,41,46,49,51,52,55,57,59,60,61^ Visual inspection of funnel plots and Begg test indicated no significant publication bias.

Headache intensity was reported in 19 studies.^22,23,29,30,31,32,33,39,40,41,46,49,51,52,55,57,59,60,61^ Heterogeneity for intensity analysis (*I^2^* = 43.5%) was comparable to the primary outcome. Compared with placebo, propranolol and cinnarizine (RoM, 0.45; 95% CI, 0.28-0.72), pregabalin (RoM, 0.57; 0.33-0.96), valproate (RoM, 0.60; 95% CI, 0.49-0.72), levetiracetam (RoM, 0.62; 95% CI, 0.50-0.77), and cinnarizine (RoM, 0.64; 95% CI, 0.54-0.76) exhibited a significant effect size on reduction of prospective migraine intensity. The forest plot and network graph are presented in Figure 3. Visual inspection of the funnel plots and Begg test did not reveal any significant publication bias (eFigure 10 in Supplement 1). The additive model suggests a higher efficacy of pregabalin with riboflavin and vitamin D3 compared with pregabalin alone (RoM, 0.31; 95% CI, 0.18-0.54; *P* < .001) or placebo (RoM, 0.12; 95% CI, 0.04-0.30; *P* < .001). Results of splitting direct and indirect evidence analysis are presented in eFigure 11 in Supplement 1. The network league table and heat map are available in eTable 5 and eFigure 12 in Supplement 1, respectively.

#### Quality of Life

**Figure 4.**
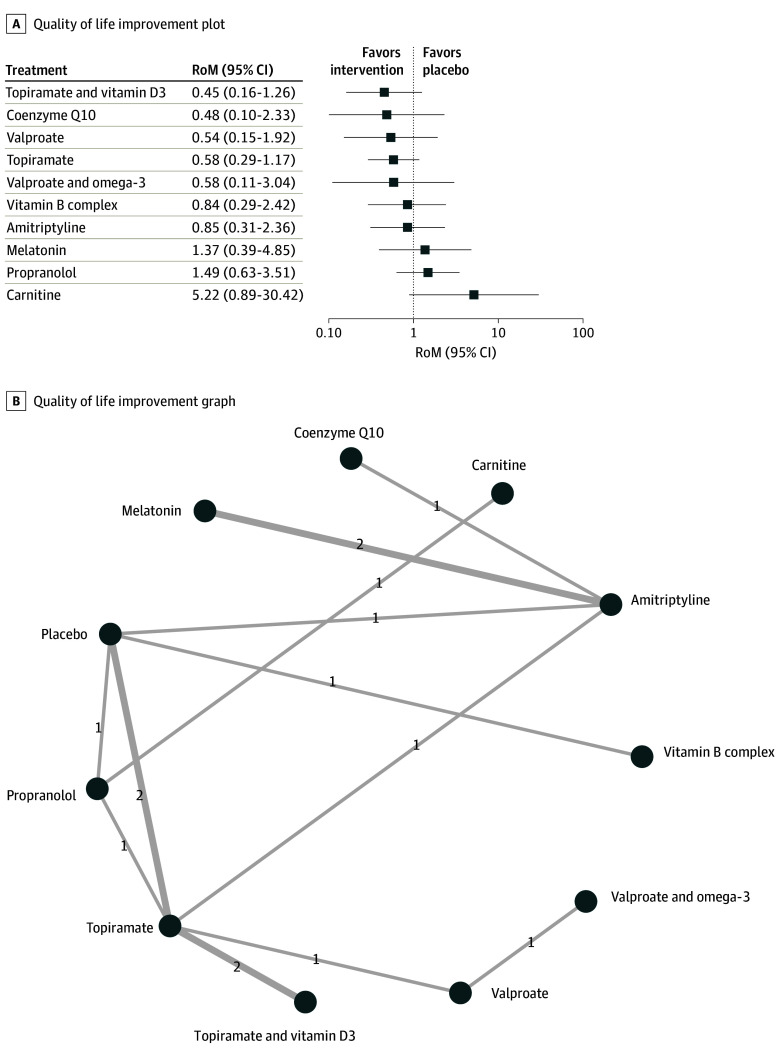
Association of Treatment With Quality of Life Forest plot (A) and network graph (B) displaying the association of various treatments with quality of life, measured using the Pediatric Migraine Disability Assessment tool. Despite evaluating multiple treatments across 13 studies,^20,30,31,32,33,35,41,42,49,52,59,60,64^ none showed significant improvements in quality of life compared with placebo. The analysis faced limitations due to the small number of studies per treatment. RoM indicates ratio of means.

Quantitative data assessing the quality of life and disability were reported in 13 studies.^20,30,31,32,33,35,41,42,49,52,59,60,64^ Notably, none of the treatments evaluated was associated with a significant reduction in disability or improvement in quality of life in the pediatric population being studied (Figure 4). However, the limited number of studies for each outcome in this analysis prevents a conclusive judgment on the effectiveness of the interventions. No significant publication bias was detected for this outcome (eFigure 13 in Supplement 1). Results from the network splitting analysis for the quality of life and disability outcomes are available in eFigure 14 in Supplement 1. The NMA heat map and details of the pairwise analysis are presented in eFigure 15 and eTable 6 in Supplement 1, respectively.

#### Headache Duration

**Figure 5.**
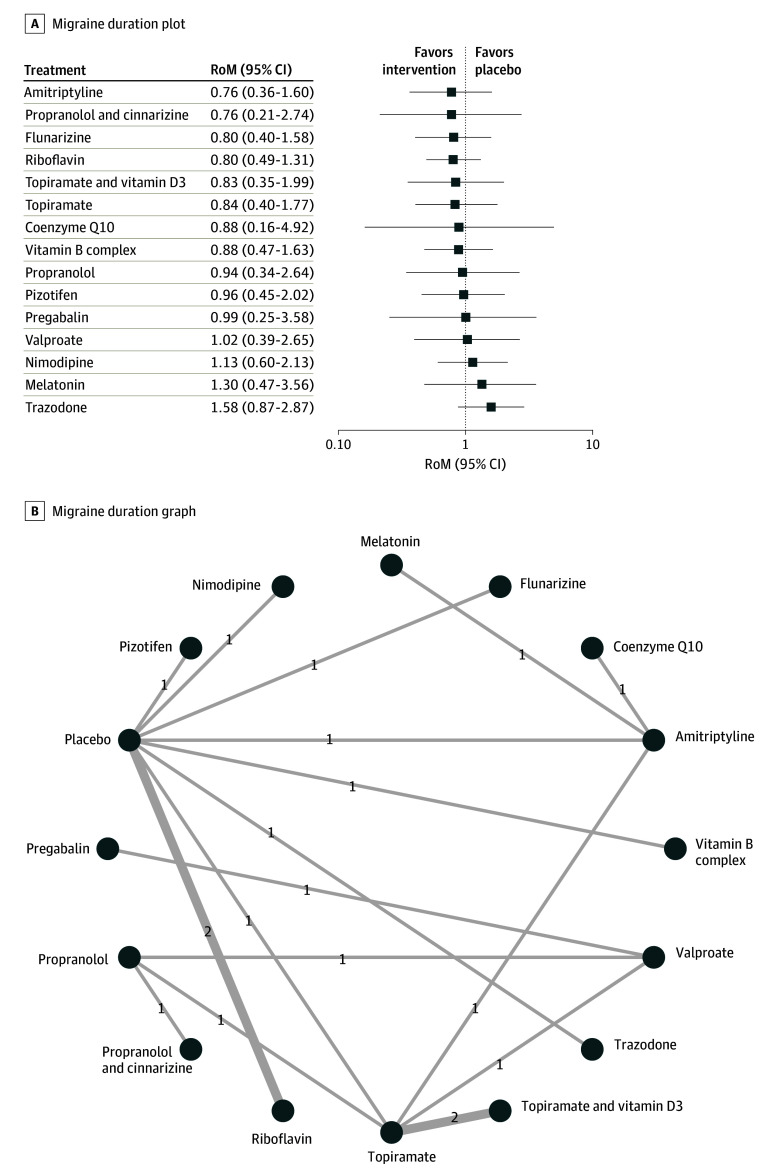
Association of Treatment With Headache Duration Forest plot (A) and network graph (B) displaying the effect of treatments on the duration of migraine attacks. Data from 17 studies^26,27,28,29,30,31,38,39,40,49,52,54,55,57,59,60,63^ covering 15 different treatments showed no significant reduction in headache duration for any of the treatments compared with placebo. Funnel plot analysis and Begg test confirmed no significant publication bias. RoM indicates ratio of means.

Similarly, a quantitative assessment of 17 studies,^26,27,28,29,30,31,38,39,40,49,52,54,55,57,59,60,63^ covering 15 treatments, showed that none of the preventive medications was associated with a reduction of the duration of migraine attacks in pediatric patients (Figure 5). There was no publication bias for this outcome (eFigure 16 in Supplement 1). eFigure 17 in Supplement 1 presents the results of the network splitting analysis for the headache duration outcome. The heat map and network league table can be found in eTable 7 and eFigure 18 in Supplement 1, respectively.

#### Safety

Adverse events were reported in 30 studies^20,21,22,23,24,26,28,29,30,31,32,33,35,36,37,44,45,46,47,48,49,51,52,54,55,56,59,60,61,64^ comprising 2486 patients. Overall, 365 patients experienced mild adverse events, and 24 patients discontinued a trial due to serious adverse events. NMA showed that amitriptyline (RR, 3.81; 95% CI, 1.41-10.32), topiramate (RR, 4.34; 95% CI, 1.60-11.75) and valproate (RR, 5.93; 95% CI, 1.93-18.23) were associated with significantly higher risks of adverse events compared with placebo (eFigure 19 and eFigure 20 in Supplement 1). No publication bias was noted (eFigure 21 in Supplement 1). Netleague for pairwise comparison of all treatments is summarized in eTable 8 in Supplement 1. Results of net splitting analysis are available in eFigure 22 in Supplement 1 and the NMA heat map for adverse events is available in eFigure 23 in Supplement 1.

## Discussion

To our knowledge, this study represents the largest NMA conducted to date on pediatric migraine preventive treatment, encompassing a substantial number of patients across multiple studies. Migraine frequency, defined as the number of attacks per month, stands as a pivotal metric in evaluating migraine severity and treatment efficacy. Our findings show that pregabalin, topiramate (both with and without vitamin D3 supplementation), flunarizine, levetiracetam, riboflavin, cinnarizine, and amitriptyline were associated with a significant reduction in migraine frequency compared with placebo.

The next outcome was 50% or greater responder rate. Flunarizine (both with and without α-lipoic acid), pregabalin, and cinnarizine showed a significant effect size on frequency reduction rates compared with placebo.

In terms of headache intensity, as measured by self-reported intensity scores, cinnarizine (both with and without propranolol), pregabalin, valproate, and levetiracetam demonstrated a significant effect size on reducing headache intensity when compared with placebo. An interesting finding was that the combination of cinnarizine and propranolol was associated with improved efficacy of cinnarizine; cinnarizine alone was associated with efficacy in reducing headache intensity by 36%, while combination of cinnarizine and propranolol was associated with a 55% reduction in intensity, and propranolol alone had no significant association with intensity.

Notably, despite the positive association with migraine frequency and headache intensity observed with these treatments, none of the included drugs were found to improve quality of life (assessed by PedMIDAS) or reduce the duration of migraine attacks. These findings underscore the complexity of pediatric migraine management and the need for further research to explore additional therapeutic avenues that may address these aspects of migraine care.

In summary, the available data indicate that the use of pregabalin and topiramate alone or in combination with high-dose vitamin B2 and D supplementation enhances objective measures of preventive efficacy. Although levetiracetam, cinnarizine, and flunarizine also showed promising results, these conclusions were drawn from individual studies, warranting cautious interpretation. Nevertheless, none of the treatments under investigation succeeded in altering patient quality of life or migraine duration. eTable 2 in Supplement 1 summarizes ranking of all treatments based on P-score for all outcomes.

It is essential to highlight that when interpreting the results of NMAs, careful consideration must be given to the quantity and quality of both direct and indirect evidence. In summary, our analysis indicates that topiramate and pregabalin exhibit a significant association with preventing pediatric migraine, supported by robust evidence from numerous studies. Conversely, while levetiracetam, cinnarizine, and flunarizine also demonstrated promising results, the conclusions drawn regarding their efficacy were primarily derived from individual studies. As such, these findings necessitate cautious interpretation and emphasize the importance of further research to substantiate their effectiveness in pediatric migraine treatment. Additionally, our analysis identified certain medications that are likely ineffective for pediatric migraine prevention, such as melatonin. Conversely, the effectiveness of other drugs warrants a more cautious interpretation, highlighting the complexity of pediatric migraine treatment and the need for further research to clarify their efficacy and safety profiles in this population.

Our research further explores an innovative analysis of combination therapies, emphasizing that the integration of supplements like vitamin D3 and riboflavin substantially augments the effectiveness of pregabalin. For example, our findings indicate that pregabalin, when used in conjunction with vitamin supplementation, may decrease the frequency of headaches by 88%. It is important to acknowledge that the results from the component NMA incorporate certain assumptions, including an additive effect between the 2 drugs. Therefore, the actual figures may be lower in clinical scenarios.^66^ We strongly recommend further investigation into these combination therapies.

This study represents an updated and expanded version of a previous NMA conducted on pediatric migraine treatment, which was published in 2020.^16^ The earlier NMA concluded that none of the drugs studied demonstrated significant effectiveness compared with placebo. In contrast, our study yielded significant results, indicating a substantial improvement in the understanding of pediatric migraine treatment efficacy.

Our study offers several notable advantages over the previous NMA.^16^ First, we included a larger number of studies, thereby enhancing the robustness and generalizability of our findings. Additionally, our methodological approach differed substantially; while the previous NMA^16^ combined all categorical variables into one variable and all continuous variables into another before measuring effect size, we conducted separate analyses for each key feature of migraine. This approach provides a more nuanced and comprehensive understanding of the efficacy of the treatments studied.

Furthermore, we employed meta-regression analysis to assess the association of baseline characteristics with treatment outcomes. Our findings suggest that differences in baseline characteristics did not significantly influence the final results, further strengthening the validity and reliability of our conclusions. The previous NMA^16^analyzed flunarizine, pregabalin, propranolol, and topiramate. The most notable difference in findings between our study and the previous NMA^16^ lies in the assessment of propranolol. While both our study and the previous study^16^ reported no effect of propranolol in preventing pediatric migraine, the earlier analysis indicated that propranolol was comparatively more effective than our study suggests.

Evidence-based research on pediatric migraine faces a major challenge, which is high placebo effect.^12^ In a randomized, double-blind, placebo-controlled clinical trial of propranolol,^41^ it was found that propranolol was effective in pediatric migraine prevention, but its effect was not significant compared with placebo. The authors stated that this finding could be because of high placebo effect among pediatric patients. This high effect was also previously reported.^10^ For instance, in a meta-analysis published in 2013,^67^ placebo was shown to effectively reduce migraine frequency among children. The previous NMA^16^ also stated that the lack of significant results could be due to the placebo effect in children, which indicates robustness of our results and also shows that all findings are somehow underestimated.

### Limitations

This study encountered several limitations. First, there may have been differences in baseline values of some outcomes (frequency, intensity, PedMIDAS, and duration), potentially compromising the validity of the results. Second, the efficacy NMA faced limitations due to some substances being tested in fewer than 100 patients, potentially leading to small-study effects influencing the observed effects. The NMA also revealed substantial heterogeneity in dosages, treatment formats, and reporting methods, which could affect both statistical and clinical outcomes.

In this systematic review, we found that Iran contributed the highest percentage of participants, accounting for approximately 44.8% of the total 3771 participants across all included studies. This was an interesting finding for us, but it also indicates a potential source of bias that needs to be addressed by researchers to ensure more balanced and generalizable results across different populations.

For future investigations, we recommend conducting randomized, placebo-controlled clinical trials on several medications, namely levetiracetam, cinnarizine, and flunarizine, because these drugs have been the subject of limited study. Our study also incorporated an analysis of combination therapy with supplements. It is important to note that the recommendations presented are based solely on statistical findings from our study and are not supported by published evidence-based studies in clinical settings. Nevertheless, we strongly advocate for further research to be conducted on these combinations.

## Conclusions

In conclusion, this comprehensive NMA sheds light on the pharmacological management of pediatric migraine, revealing that treatments like pregabalin, topiramate (with and without vitamin D3 supplementation), levetiracetam, flunarizine, riboflavin, amitriptyline, and cinnarizine may reduce migraine frequency in pediatric patients. However, these treatments did not improve quality of life or reduce the duration of migraine attacks, highlighting the need for further research to develop more comprehensive therapeutic strategies. The study underscores the potential benefits of combination therapies, particularly those involving vitamin supplementation, and emphasizes the importance of larger, randomized clinical trials to confirm these findings and explore new avenues for enhancing care in pediatric migraine management.

## Notes

Supplement 1. **eAppendix.** Search Terms **eFigure 1.** PRISMA Flow Diagram Presenting Study Selection Process **eTable 1.** Characteristics of Included Studies **eFigure 2.** Details of Risk of Bias Assessment for Each Included Study Based on Cochrane Risk of Bias Tool Version 2 **eFigure 3.** Overall Risk of Bias Percentage for Different Domains Based on Cochrane Risk of Bias Tool Version 2 **eTable 2.** P-scores of Efficacies of All Study Interventions Included for Each Outcome **eFigure 4.** Funnel Plot For Frequency **eTable 3.** Network League for Headache Frequency **eFigure 5.** Network Splitting Analysis of Headache Frequency **eFigure 6.** Network Meta-Analysis Heat Map for Headache Frequency **eFigure 7.** Funnel Plot for 50% Headache Frequency Reduction Rate\ **eTable 4.** Network League for 50% Headache Reduction Rate **eFigure 8.** Net Splitting Analysis for 50% Headache Frequency Reduction Rate **eFigure 9.** Network Meta-Analysis Heat Map for 50% Headache Frequency Reduction Rate **eFigure 10.** Funnel Plot for Migraine Intensity **eFigure 11.** Network Splitting Analysis for Headache Intensity **eTable 5.** Network League Table for Headache Intensity **eFigure 12.** Network Meta-Analysis Heat Map for Headache Intensity **eFigure 13.** Funnel Plot for Quality-of-Life Outcome **eFigure 14.** Net Splitting Analysis for Quality of Life **eFigure 15.** Network Meta-Analysis Heat Map for Quality of Life **eTable 6.** Network League Table for Quality of Life **eFigure 16.** Funnel Plot for Headache Duration **eFigure 17.** Network Splitting Analysis for Headache Duration **eTable 7.** Network League Table for Headache Duration **eFigure 18.** Network Meta-Analysis Heat Map for Headache Duration **eFigure 19.** Forest Plot of Adverse Events of Each Intervention Compared With Placebo **eFigure 20.** Network Graph for Adverse Events **eFigure 21.** Funnel Plot for Safety **eTable 8.** Network League Table for Adverse Events **eFigure 22.** Net Splitting Analysis for Adverse Events **eFigure 23.** Network Meta-Analysis Heat Map for Adverse Events
eReferences.

Supplement 2.
Data Sharing Statement
