# Supplementary material for: Preventive Medications in Pediatric Migraine: A Network Meta-Analysis
Source: JAMA Netw Open. 2024 Oct 10;7(10):e2438666. doi: 10.1001/jamanetworkopen.2024.38666 (PMC11581497; doi:10.1001/jamanetworkopen.2024.38666)
Supplement: Supplement 1. — eAppendix. Search Terms eFigure 1. PRISMA Flow Diagram Presenting Study Selection Process eTable 1. Characteristics of Included Studies eFigure 2. Details of Risk of Bias Assessment for Each Included Study Based on Cochrane Risk of Bias Tool Version 2 eFigure 3. Overall Risk of Bias Percentage for Different Domains Based on Cochrane Risk of Bias Tool Version 2 eTable 2. P-scores of Efficacies of All Study Interventions Included for Each Outcome eFigure 4. Funnel Plot For Frequency eTable 3. Network League for Headache Frequency eFigure 5. Network Splitting Analysis of Headache Frequency eFigure 6. Network Meta-Analysis Heat Map for Headache Frequency eFigure 7. Funnel Plot for 50% Headache Frequency Reduction Rate\ eTable 4. Network League for 50% Headache Reduction Rate eFigure 8. Net Splitting Analysis for 50% Headache Frequency Reduction Rate eFigure 9. Network Meta-Analysis Heat Map for 50% Headache Frequency Reduction Rate eFigure 10. Funnel Plot for Migraine Intensity eFigure 11. Network Splitting Analysis for Headache Intensity eTable 5. Network League Table for Headache Intensity eFigure 12. Network Meta-Analysis Heat Map for Headache Intensity eFigure 13. Funnel Plot for Quality-of-Life Outcome eFigure 14. Net Splitting Analysis for Quality of Life eFigure 15. Network Meta-Analysis Heat Map for Quality of Life eTable 6. Network League Table for Quality of Life eFigure 16. Funnel Plot for Headache Duration eFigure 17. Network Splitting Analysis for Headache Duration eTable 7. Network League Table for Headache Duration eFigure 18. Network Meta-Analysis Heat Map for Headache Duration eFigure 19. Forest Plot of Adverse Events of Each Intervention Compared With Placebo eFigure 20. Network Graph for Adverse Events eFigure 21. Funnel Plot for Safety eTable 8. Network League Table for Adverse Events eFigure 22. Net Splitting Analysis for Adverse Events eFigure 23. Network Meta-Analysis Heat Map for Adverse Events eReferences. [file jamanetwopen-e2438666-s001.pdf]

Supplemental Online Content

Kohandel Gargari O, Aghajanian S, Togha M, et al. Effectiveness of preventive medications in pediatric medicine: a network meta-analysis. *JAMA Netw. Open*. 2024;7(9):e2438666.
doi:10.1001/jamanetworkopen.2024.38666

eAppendix. Search Terms

eFigure 1. PRISMA Flow Diagram Presenting Study Selection Process

eTable 1. Characteristics of Included Studies

eFigure 2. Details of Risk of Bias Assessment for Each Included Study Based on Cochrane Risk of Bias Tool Version 2

eFigure 3. Overall Risk of Bias Percentage for Different Domains Based on Cochrane Risk of Bias Tool Version 2

eTable 2. P-scores of Efficacies of All Study Interventions Included for Each Outcome

eFigure 4. Funnel Plot For Frequency

eTable 3. Network League for Headache Frequency

eFigure 5. Network Splitting Analysis of headache Frequency

eFigure 6. Network Meta-Analysis Heatmap for Headache Frequency

eFigure 7. Funnel Plot for 50% Headache Frequency Reduction Rate\

eTable 4. Network League for 50% Headache Reduction Rate

eFigure 8. Net Splitting Analysis for 50% Headache Frequency Reduction Rate

eFigure 9. Network Meta-Analysis Heatmap for 50% Headache Frequency Reduction Rate

eFigure 10. Funnel Plot for Migraine Intensity

eFigure 11. Network Splitting Analysis for Headache Intensity

eTable 5. Network League Table for Headache Intensity

eFigure 12. Network Meta-Analysis Heatmap for Headache Intensity

eFigure 13. Funnel Plot for Quality-of-Life Outcome

eFigure 14. Net Splitting Analysis for Quality of Life

eFigure 15. Network Meta-Analysis Heatmap for Quality of Life

eTable 6. Network League Table for Quality of Life

eFigure 16. Funnel Plot for Headache Duration

eFigure 17. Network Splitting Analysis for Headache Duration

eTable 7. Network League Table for Headache Duration

eFigure 18. Network Meta-Analysis Heatmap for Headache Duration

© 2024 Kohandel Gargari O et al. *JAMA Network Open.*

eFigure 19. Forest Plot of Adverse Events of Each Intervention Compared With Placebo

eFigure 20. Network Graph for Adverse Events

eFigure 21. Funnel Plot for Safety

eTable 8. Network League Table for Adverse Events

eFigure 22. Net Splitting Analysis for Adverse Events

eFigure 23. Network Meta-Analysis Heatmap for Adverse Events

eReferences.

This supplemental material has been provided by the authors to give readers additional information about their work.

© 2024 Kohandel Gargari O et al. *JAMA Network Open.*

eAppendix. Search Terms

Terms within arms were searched using “OR” operator and arms were searched with “AND.”

Arm1
randomized controlled trial
trial*
Random*
Placebo*
therapy
treatment
prevention
Prophylaxis
drug
group*
arm
control
Arm2
migraine
headache
Cephalalgia
Arm3
child*
pediatric*
paediatric*
infant*
juvenile*
adolescen*
developmental age

© 2024 Kohandel Gargari O et al. *JAMA Network Open.*

eFigure 1. PRISMA Flow Diagram Presenting Study Selection Process

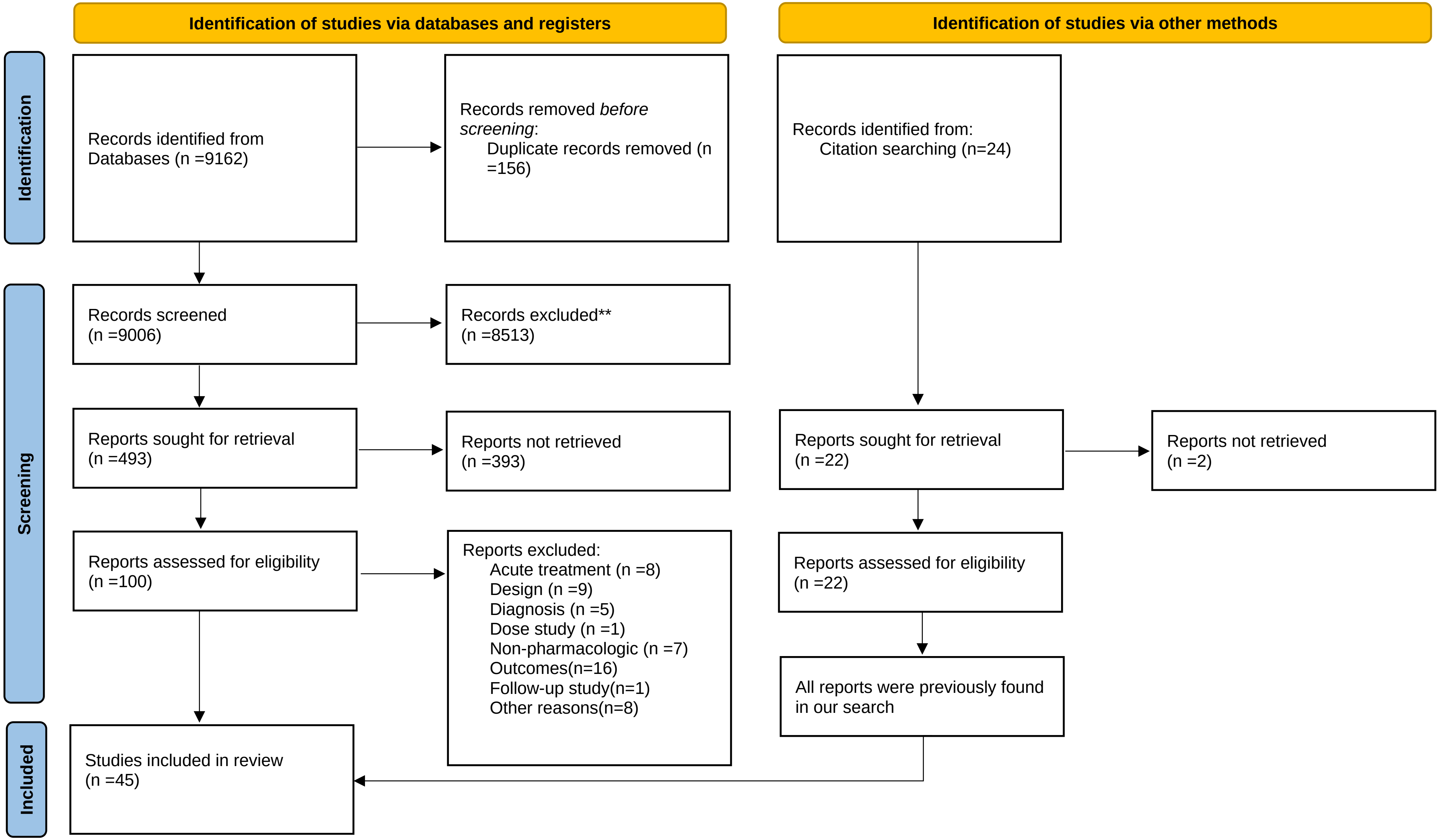

© 2024 Kohandel Gargari O et al. *JAMA Network Open.*

eTable 1. Characteristics of Included Studies

This table outlines the key characteristics of the included studies on migraine interventions, including the study author and year, country of origin, total number of participants, migraine characteristics, diagnostic criteria used, intervention details, measured outcomes, overall follow-up duration, and the assessed risk of bias. Outcomes are categorized as follows: Frequency - the number of migraine attacks per month after treatment; 50% responder rate - defined as the number of patients with at least a 50% reduction in headache frequency after treatment compared to baseline; Intensity- self-reported headache intensity, a numeric assessment of pain intensity from 0 to 10; Quality of Life/Disability - disability produced by migraine, measured by the PedMIDAS tool, a six-question instrument developed to assess the disability caused by migraines in school-age children and adolescents; Duration- the length of each individual headache attack, measured in minutes or hours. For studies with multiple endpoints, the last follow-up time was selected for analysis. Additionally, the overall number of adverse events was monitored to assess safety. Abbreviations: ICHD - International Classification of Headache Disorders; NR - Not Reported; DVPX ER - Divalproex Extended Release; ALA - Alpha-Lipoic Acid; IU - International Units; PedMIDAS - Pediatric Migraine Disability Assessment Score.

Study	Year	Country	Total participants	Migraine characteristics	Diagnostic criteria	Interventions			Outcomes					Overall follow-up	Risk of bias
						1	2	3	Frequency	50% Responder rate	Intensity	QoL/Disability	Duration		
Amanat (1)	2020	Iran	149	W & W/O Aura	ICHD-2	Cinnarizine (6-12 years: 37.5 mg/day; 12–17 years: 50 mg/day divided into two doses	Sodium Valproate (15 mg/kg/day divided in two dose)	Placebo	✓	✓	✓			12 Weeks	Low
Amini (2)	2021	Iran	56	W & W/O Aura	ICHD-3	Carnitine (50mg/kg/day divided into two doses)	Propranolol (3 mg/kg daily)	-	✓			✓		12 Weeks	Low
Apostol (3)	2008	USA	299	NR	ICHD-2	DVPX ER 250 mg/daily	Placebo			✓				3 Month	Low
Ashrafi (4)	2005	Iran	120	W/O Aura	ICHD-1	Sodium Valproate (starting dose: 10 mg/kg/day in two divided doses; increased up to 40 mg/kg/day)	Propranolol (1–3 mg/kg/day divided in two doses)	-	✓	✓				2 Months	Low
Ashrafi (5)	2014	Iran	40	W & W/O Aura	ICHD-2	Cinnarizine (4–11 years: 37.5 mg/day for 12 weeks; 12 17–years: 50 mg/day for 12 weeks)	Topiramate (50 mg/kg/day for 12 weeks)	-	✓	✓	✓			12 Weeks	Low
Ashrafi (6)	2014	Iran	62	W & W/O Aura	ICHD-2	Cinnarizine (below 30kg: 1.5mg/kg/day for 12 weeks; Above 30kg: 50mg/day for 12 weeks)	Placebo	-	✓	✓	✓			3 Months	Low

© 2024 Kohandel Gargari O et al. *JAMA Network Open.*

Bakhshandeh Bali (7)	2015	Iran	91	W & W/O Aura	ICHD-2	Pregabalin (50 to 75 mg/day for at least 8 weeks)	Propranolol (10-20mg/day in two divided doses)	-	✓	✓				2 Months	Low
Battistella (8)	1990	Italy	37	W & W/O Aura	ICHD-1	Nimodipine (10 to 20 mg three times/day)	Placebo	-	✓				✓	3 Months	Medium
Battistella (9)	1993	Italy	40	W/O Aura	ICHD-1	Trazodone (1 mg/kg/day)	Placebo	-	✓				✓	12 Weeks	Medium
Bidabadi (10)	2010	Iran	60	W/O Aura	ICHD-2	Propranolol (3mg/kg/day in two divided doses then 2 mg/kg/day)	Sodium Valproate (30 mg/kg/day in 2 divided doses adjusted to 15 mg/kg/day after 1 month)	-	✓	✓			✓	4 Months	Low
Bruijn (11)	2010	Netherlands	42	W & W/O Aura	ICHD-2	Riboflavin (50mg/day for 16 weeks)	Placebo (carotene 100/day for 16 weeks)	-	✓		✓		✓	4 Months	Low
Dalrymple (12)	2017	USA	328	NR	NR	Topiramate (2 mg/kg/day)	Amitriptyline (1 mg/kg/day)	Placebo		✓				3 Month	Low
Elmala (13)	2022	Saudi Arabia	60	W & W/O Aura	ICHD-3	Topiramate + Vitamin D3 (2 mg/kg in 2 divided doses daily + 5000 IU daily)	Topiramate (2 mg/kg in 2 divided doses daily + Placebo)	-	✓	✓	✓	✓	✓	4 Months	Low
Fallah (14)	2013	Iran	100	W & W/O Aura	ICHD-2	Propranolol (1 mg/kg daily)	Topiramate (3 mg/kg daily)	-	✓	✓	✓	✓		12 Weeks	Medium
Fallah (15)	2018	Iran	80	W & W/O Aura	ICHD-2	Melatonin (0.3 mg/kg)	Amitriptyline (0.1 mg/kg)	-	✓	✓	✓	✓		90 Days	Medium
Fallah (16)	2020	Iran	57	W & W/O Aura	ICHD-2	Topiramate + Vitamin D3 (2 mg/kg/day + 500,000 IU pearl/week)	Topiramate (2 mg/kg in 2 divided doses daily)	-	✓	✓	✓	✓	✓	3 months	Low
Fayyazi (17)	2016	Iran	25	W & W/O Aura	ICHD	Valproate + Omega-3 (20 mg/ kg+ 1g of fish oil)	Valproate (20 mg/ kg)	-	✓			✓		2 Months	Low
Fayyazi (18)	2022	Iran	30	W & W/O Aura	Pediatrician	Melatonin + Propranolol (3 mg/day + 1mg/kg/day)	Propranolol (1 mg/kg/day)	-	✓					4 Months	Low
Gelfand (19)	2023	USA	42	W & W/O Aura	ICHD-3	Melatonin (3mg/day and 6 mg/day)	Placebo	-	✓					16 Weeks	Low

© 2024 Kohandel Gargari O et al. *JAMA Network Open.*

Ghazavi (20)	2019	Iran	60	W & W/O Aura	ICHD-3	Levetiracetam (10 mg/kg/day (divided in two doses q 12 h) and increased to 20 mg/kg/ day (divided in two doses q 12 h) after one week	Amitriptyline: starting dose was 0.25 mg/kg/ day (bedtime), and this was increased by 0.25 mg/kg/day weekly to final dose of 1 mg/kg/day (bedtime)	-		✓				3 Months	Low
Gibler (21)	2023	USA	175	W/O Aura	ICHD-3	Amitriptyline	Topiramate	Placebo	✓				✓	28 Days	Low
Gillies (22)	1986	UK	39	W & W/O Aura	-	Pizotifen (0.5 mg three times/day)	Placebo	-	✓				✓	3 Months	Low
Jafari (23)	2023	Iran	56	-	ICHD-3	Sodium Valproate	Pregabalin	-	✓	✓	✓		✓	4 Months	Low
Katibeh (24)	2020	Iran	90	W & W/O Aura	ICHD-3	Propranolol + Cinnarizine	Propranolol	-	✓		✓		✓	2 Months	Low
Keerthana (25)	2022	India	23	W/O Aura	ICHD-3	Propranolol (1-3mg/kg/day in two divided doses)	Placebo	-	✓	✓	✓	✓		3 Months	Low
Lakshmi (26)	2007	India	44	W & W/O Aura	ICHD-2	Topiramate (Titrated to 100mg)	Placebo	-	✓	✓		✓		4 Months	Low
Lewis (27)	2009	USA	85	W & W/O Aura	ICHD-2	Topiramate (100 mg/day)	Placebo	-	✓					12 Weeks	Medium
Ludvigsson (28)	1974	Sweden	32	NR	Ad hoc Committee	Propranolol: 60 mg/ day (weighting less than 35) and 120 mg/day (for more than 35 mg)	Placebo			✓				3 Months	Low
MacLennan (29)	2008	Australia	48	W & W/O Aura	ICHD-2	Riboflavin: 200 mg/day	Placebo			✓				12 weeks	Medium
Montazerlotfelahi (30)	2019	Iran	68	W & W/O Aura	ICHD-2	Levetiracetam (20 mg/kg/day divided into two doses up to 40 mg/kg/day)	Placebo	-	✓	✓	✓			3 Months	Medium
Oelkers-Ax (31)	2008	Germany	39	W & W/O Aura	ICHD-1	Butterbur root extract (50-100 mg/day for 8 weeks)	Placebo	-	✓	✓				6 Months	Low

© 2024 Kohandel Gargari O et al. *JAMA Network Open.*

Powers (32)	2017	USA	205	W & W/O Aura	ICHD-2	Amitriptyline (1 mg/kg/day)	Topiramate (2 mg/kg/day)	Placeb o	✓	✓		✓		24 Weeks	Low
Puliappadamb (33)	2023	India	60	NR	ICHD-3	flunarizine (5 mg/day) + ALA (300 mg/day)	flunarizine (5 mg/day			✓				12 weeks	Low
Sadeghvand (34)	2023	Iran	90	W & W/O Aura	ICHD-2	Vitamin B Complex (one capsule once daily)	Placebo	-	✓		✓	✓	✓	6 Month s	Low
Santucci (35)	1986	Italy	27	-	Ad Hoc Committee	5-Hydroxytryptopha n (5mg/kg/day)	Placebo	-	✓					3 Month s	Low
Sezer (36)	2013	Turkey	57	-	ICHD-2	Amitriptyline (0.5 mg/kg/day)	Topiramate (25 mg/day increasing up to 100 mg/day)	-	✓	✓	✓			4 Month s	Low
Shahnawaz (37)	2019	India	90	W & W/O Aura	ICHD-2	Melatonin (0.3mg/kg for 12 weeks)	Amitriptyline (1mg/kg/day)	-	✓	✓	✓	✓	✓	3 Month s	Low
Slater (38)	2011	USA	120	W & W/O Aura	ICHD-2	Coenzyme Q10 (100 mg/day)	Placebo	-	✓					16 Weeks	Low
Sorge (39)	1985	Italy	48	W & W/O Aura	Valquist's criteria and clinical observatio n	Flunarizine (5mg/day)	Placebo	-	✓	✓			✓	3 Month s	Low
Talebian (40)	2018	Iran	90	W & W/O Aura	ICHD-2	Riboflavin (200 mg for 12 weeks)	Placebo	-	✓	✓	✓		✓	3 Month s	Low
Togha (41)	2011	Iran	113	W & W/O Aura	ICHD-2	Cinnarizine (37.5-50mg/day in two divided doses)	Propranolol (1 mg/kg/day)	-	✓	✓				3 Month s	Low

© 2024 Kohandel Gargari O et al. *JAMA Network Open.*

Tonekaboni (42)	2013	Iran	78	-	IHSC 2004	Topiramate (50-100mg/day)	Propranolol (20-80mg/day)	-	✓		✓		✓	4 Months	Low
Winner (43)	2005	USA	162	W & W/O Aura	IHS	Topiramate: 15 mg/day (week 1), increased to 30 mg/day (week 2), and further increased to 50 mg/day at week 3	Placebo			✓				12 weeks	Low
Yadav (44)	2017	India	82	W/O Aura	ICHD-2	Sodium Valproate (10-20mg/kg for 12 weeks)	Topiramate (1-2mg/kg for 12 weeks)	-		✓	✓	✓	✓	3 Months	Medium
Yaghini (45)	2022	Iran	72	W & W/O Aura	ICHD-3	Coenzyme Q10 (30-60 mg)	Amitriptyline (1-2 mg/kg)	-	✓		✓	✓	✓	3 Months	Low

© 2024 Kohandel Gargari O et al. *JAMA Network Open.*

eFigure 2. Details of Risk of Bias Assessment for Each Included Study Based on Cochrane Risk of Bias Tool Version 2

Study	D1	D2	D3	D4	D5	Overall
Ludvigson (1974)	+	+	+	+	+	+
Ahrafi (2014)	+	+	+	+	+	+
Amanat (2020)	+	+	+	+	+	+
Amini (2021)	+	+	+	+	+	+
Apostol (2008)	+	+	+	+	+	+
Ashrafi (2005)	+	+	+	+	+	+
Ashrafi (2014)	+	+	+	+	+	+
Bakhshandeh Bali (2015)	+	+	+	+	+	+
Battistella (1990)	+	+	X	+	+	-
Battistella (1993)	+	+	+	+	X	-
Bidabadi (2010)	-	+	+	+	+	+
Brujin (2010)	+	+	+	+	+	+
Dalrymple (2017)	+	+	+	+	+	+
Elmala (2022)	+	+	+	+	+	+
Fallah (2020)	-	+	+	+	+	+
Fallah R. (2013)	X	+	+	+	+	-
Fallah R. (2018)	X	+	+	+	+	-
Fayyazi (2016)	+	+	+	+	+	+
Fayyazi (2022)	+	+	+	+	+	+
Gallelli (2013)	-	+	+	+	+	+
Gelfand (2017)	+	+	+	+	+	+
Gelfand (2023)	+	+	-	+	+	+
Ghazavi (2019)	+	+	+	+	+	+
Gibler (2023)	+	+	+	+	+	+
Gillies (1986)	+	+	+	+	+	+
Jafari (2023)	+	+	-	+	+	+
Katibeh (2020)	+	+	+	+	+	+
Keerthana (2022)	+	+	+	+	+	+
Lakshmi (2007)	+	+	+	+	+	+
Lewis (2009)	+	+	X	+	+	-
MacLennan (2008)	+	+	X	+	+	-
Montazerlotfelahi (2019)	+	+	-	+	+	+
Oelkers-Ax (2008)	+	+	X	+	+	-
Powers S. (2017)	+	+	+	+	+	+
Powers S. (2021)	+	+	+	+	+	+
Sadeghvand (2023)	+	+	+	+	+	+
Santucci (1986)	+	+	-	+	+	+
Sezer (2013)	+	+	+	+	+	+
Shahnawaz (2019)	+	+	+	+	+	+
Slater (2011)	+	+	+	+	+	+
Sorge (1985)	+	+	+	+	+	+
Talebian (2018)	+	+	+	+	+	+
Togha (2011)	+	+	+	+	+	+
Tonekaboni (2013)	+	+	+	+	+	+
Wang (2003)	+	+	+	+	+	+
Winner (2020)	+	+	+	+	+	+
Yadav (2017)	+	+	-	+	-	-
Yaghini (2022)	+	+	+	+	+	+

Risk of bias domains

Domains:
D1: Bias arising from the randomization process.
D2: Bias due to deviations from intended intervention.
D3: Bias due to missing outcome data.
D4: Bias in measurement of the outcome.
D5: Bias in selection of the reported result.

Judgement
X High
- Some concerns
+ Low

© 2024 Kohandel Gargari O et al. *JAMA Network Open.*

eFigure 3. Overall Risk of Bias Percentage for Different Domains Based on Cochrane Risk of Bias Tool Version 2

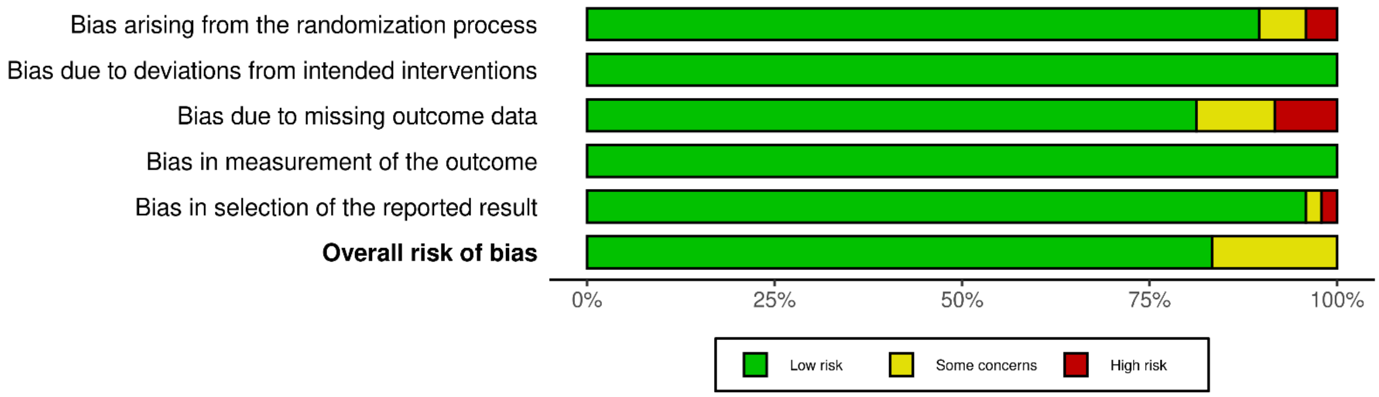

© 2024 Kohandel Gargari O et al. *JAMA Network Open.*

eTable 2. P-Scores of Efficacies of All Study Interventions Included for Each Outcome
This table presents the P-scores of the efficacies of all study interventions included for each outcome. P-scores provide a measure of the extent to which an intervention is effective, with higher P-scores indicating greater efficacy

**Headache frequency**		**50% responder rate**		**Headache intensity**		**Quality of life & Disability**		**Migraine duration**	
**Treatment**	**P-score**	**Treatment**	**P-score**	**Treatment**	**P-score**	**Treatment**	**P-score**	**Treatment**	**P-score**
Pregabalin	0.9140	Flunarizine + ALA	0.9954	Propranolol + Cinnarizine	0.9560	Topiramate + Vitamin D3	0.7973	Amitriptyline	0.6760
Topiramate + Vitamin D3	0.8904	Flunarizine	0.9007	Valproate	0.8066	CoenzymeQ10	0.7362	Riboflavin	0.6353
Levetiracetam	0.8601	Pregabalin	0.7779	Pregabalin	0.7887	Valproate	0.6995	Propranolol + Cinnarizine	0.6297
Flunarizine	0.8583	Topiramate + VitD3	0.6687	Levetiracetam	0.7350	Topiramate	0.6937	Flunarizine	0.6217
Riboflavin	0.8307	Cinnarizine	0.6266	Cinnarizine	0.7092	Valproate + Omega-3	0.6426	Topiramate	0.5988
Cinnarizine	0.7021	Levetiracetam	0.5649	Topiramate + Vitamin D3	0.5951	Vitamin B Complex	0.5016	Topiramate + Vitamin D3	0.5972
Melatonin + Propranolol	0.6613	Riboflavin	0.5115	Topiramate	0.5660	Amitriptyline	0.4951	Vitamin B Complex	0.5558
Topiramate	0.6374	Valproate	0.4585	Amitriptyline	0.4475	Placebo	0.3963	CoenzymeQ10	0.5341
Amitriptyline	0.6093	Topiramate	0.4179	Vitamin B Complex	0.4210	Propranolol	0.2698	Propranolol	0.4925
Vitamin B Complex	0.4745	Propranolol	0.4027	Propranolol	0.3362	Melatonin	0.2326	Pizotifen	0.4914
Pizotifen	0.4724	Butterbur root extract	0.3274	CoenzymeQ10	0.2804	Carnitine	0.0353	Pregabalin	0.4710
Valproate + Omega-3	0.4535	Amitriptyline	0.3264	Placebo	0.2002			Placebo	0.4439
Valproate	0.3853	Divalproex	0.2010	Riboflavin	0.1245			Valproate	0.4330
Placebo	0.3385	Placebo	0.1971	Melatonin	0.0336			Nimodipine	0.3682
Propranolol	0.3382	Melatonin	0.1233					Melatonin	0.2906
5-Hydroxytryptamine	0.3240							Trazodone	0.1608
CoenzymeQ10	0.3105								
Propranolol + Cinnarizine	0.2990								
Carnitine	0.2980								
Nimodipine	0.2633								
Butterbur root extract	0.2577								
Trazodone	0.1982								
Melatonin	0.1234								

© 2024 Kohandel Gargari O et al. *JAMA Network Open.*

eFigure 4. Funnel Plot For Frequency

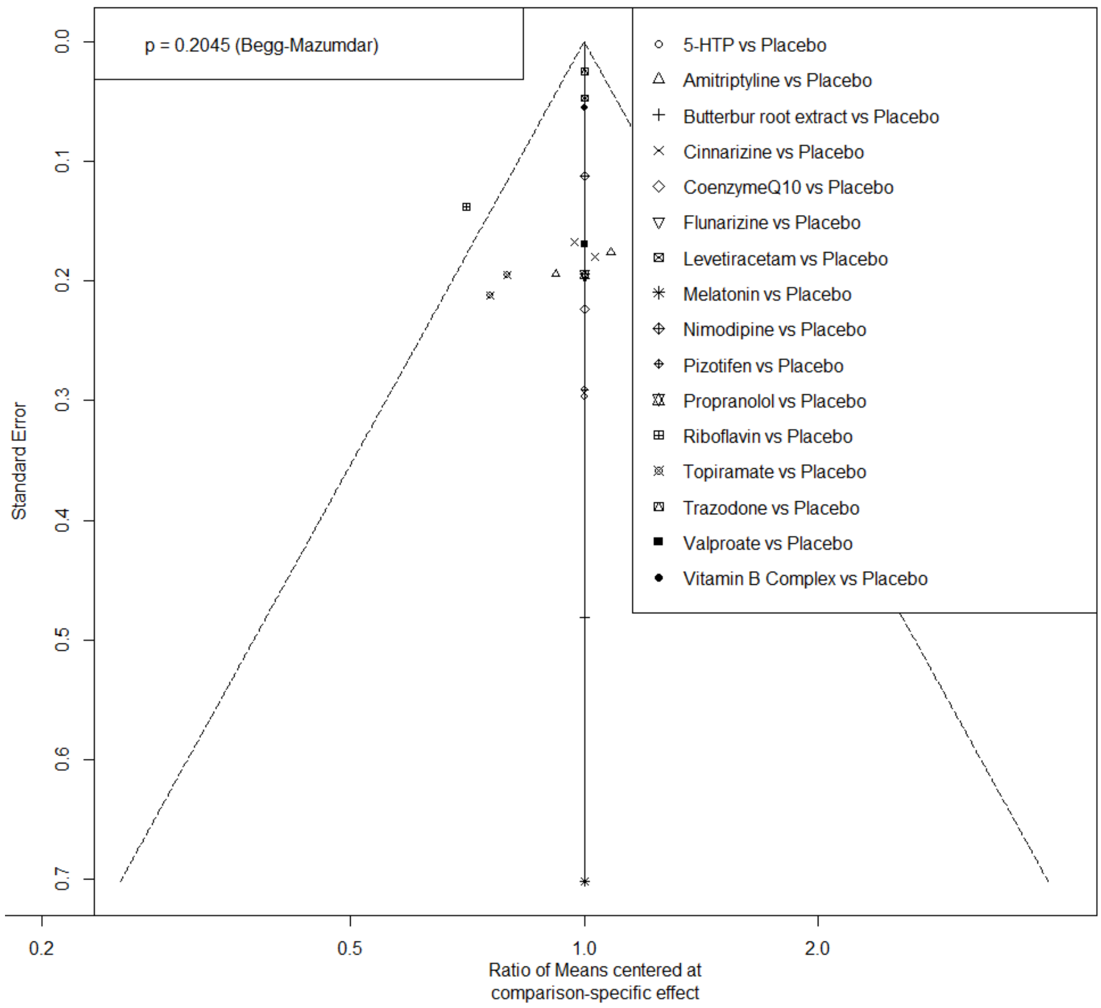

© 2024 Kohandel Gargari O et al. *JAMA Network Open.*

eTable 3. Network League Table for Headache Frequency

This table presents the network league table for headache frequency, comparing the relative efficacy of different interventions. The table displays the ratio of means in headache frequency between each pair of interventions, with corresponding 95% confidence intervals (CIs)

1	5-HTP	.	.	.	.	.	.	.	.	.	.	.	1.10 (0.54; 2.26)	.	.	.	.	.	.	.	.	.	.
2	1.52 (0.70; 3.29)	Amitriptyline	.	.	.	0.92 (0.46; 1.87)	.	.	0.52 (0.37; 0.75)	.	.	.	0.83 (0.56; 1.23)	.	.	.	.	0.93 (0.70; 1.22)	.	.	.	.	.
3	0.88 (0.25; 3.09)	0.58 (0.20; 1.69)	Butterbur root extract	.	.	.	.	.	.	.	.	.	1.25 (0.45; 3.52)	.	.	.	.	.	.	.	.	.	.
4	1.02 (0.39; 2.65)	0.67 (0.35; 1.30)	1.16 (0.35; 3.89)	Carnitine	.	.	.	.	.	.	.	.	.	.	1.08 (0.63; 1.84)	.	.	.	.	.	.	.	.
5	1.73 (0.78; 3.81)	1.14 (0.75; 1.73)	1.97 (0.67; 5.83)	1.69 (0.87; 3.28)	Cinnarizine	.	.	.	.	.	.	.	0.52 (0.36; 0.77)	.	1.21 (0.52; 2.81)	.	.	0.74 (0.31; 1.76)	.	.	0.82 (0.47; 1.42)	.	.
6	1.07 (0.45; 2.53)	0.70 (0.43; 1.15)	1.22 (0.39; 3.80)	1.04 (0.48; 2.27)	0.62 (0.35; 1.10)	CoenzymeQ10	.	.	.	.	.	.	1.26 (0.69; 2.32)	.	.	.	.	.	.	.	.	.	.
7	2.41 (0.96; 6.05)	1.59 (0.84; 3.02)	2.75 (0.84; 8.96)	2.36 (1.01; 5.50)	1.40 (0.72; 2.70)	2.26 (1.07; 4.76)	Flunarizine	.	.	.	.	.	0.46 (0.26; 0.81)	.	.	.	.	.	.	.	.	.	.
8	2.35 (1.02; 5.45)	1.55 (0.92; 2.62)	2.68 (0.87; 8.23)	2.30 (1.08; 4.92)	1.36 (0.79; 2.35)	2.20 (1.16; 4.20)	0.98 (0.48; 2.00)	Levetiracetam	.	.	.	.	0.47 (0.30; 0.72)	.	.	.	.	.	.	.	.	.	.
9	0.79 (0.34; 1.84)	0.52 (0.37; 0.74)	0.90 (0.29; 2.78)	0.78 (0.37; 1.62)	0.46 (0.27; 0.78)	0.74 (0.41; 1.34)	0.33 (0.16; 0.68)	0.34 (0.18; 0.62)	Melatonin	.	.	.	1.49 (0.35; 6.28)	.	.	.	.	.	.	.	.	.	.
10	1.74 (0.57; 5.30)	1.14 (0.47; 2.76)	1.98 (0.52; 7.56)	1.70 (0.65; 4.43)	1.00 (0.41; 2.43)	1.62 (0.62; 4.29)	0.72 (0.26; 2.01)	0.74 (0.28; 1.92)	2.19 (0.85; 5.61)	Melatonin + Propranolol	.	.	.	.	0.63 (0.29; 1.40)	.	.	.	.	.	.	.	.
11	0.98 (0.41; 2.33)	0.65 (0.37; 1.13)	1.12 (0.36; 3.49)	0.96 (0.44; 2.11)	0.57 (0.32; 1.01)	0.92 (0.47; 1.81)	0.41 (0.19; 0.86)	0.42 (0.22; 0.79)	1.24 (0.65; 2.37)	0.57 (0.21; 1.51)	Nimodipine	.	1.12 (0.70; 1.80)	.	.	.	.	.	.	.	.	.	.
12	1.29 (0.47; 3.55)	0.85 (0.40; 1.84)	1.47 (0.42; 5.16)	1.26 (0.49; 3.25)	0.75 (0.34; 1.64)	1.21 (0.51; 2.85)	0.54 (0.22; 1.33)	0.55 (0.24; 1.26)	1.63 (0.71; 3.76)	0.74 (0.25; 2.26)	1.32 (0.56; 3.10)	Pizotifen	0.85 (0.42; 1.73)	.	.	.	.	.	.	.	.	.	.
13	1.10 (0.54; 2.26)	0.73 (0.54; 0.97)	1.25 (0.45; 3.52)	1.08 (0.58; 2.01)	0.64 (0.46; 0.88)	1.03 (0.64; 1.66)	0.46 (0.26; 0.81)	0.47 (0.30; 0.72)	1.39 (0.90; 2.15)	0.63 (0.27; 1.49)	1.12 (0.70; 1.80)	0.85 (0.42; 1.73)	Placebo	.	0.66 (0.37; 1.16)	.	2.01 (1.31; 3.08)	1.40 (1.05; 1.86)	.	0.82 (0.53; 1.25)	1.62 (0.94; 2.77)	.	1.17 (0.76; 1.82)
14	2.92 (1.04; 8.19)	1.93 (0.89; 4.18)	3.33 (0.94; 11.86)	2.86 (1.18; 6.96)	1.69 (0.79; 3.62)	2.74 (1.15; 6.54)	1.21 (0.48; 3.08)	1.24 (0.53; 2.92)	3.69 (1.59; 8.56)	1.68 (0.58; 4.88)	2.98 (1.24; 7.16)	2.26 (0.81; 6.29)	2.66 (1.27; 5.55)	Pregabalin	0.53 (0.16; 1.78)	.	.	.	.	.	0.34 (0.16; 0.76)	.	.
15	1.10 (0.50; 2.41)	0.73 (0.50; 1.06)	1.25 (0.43; 3.69)	1.08 (0.63; 1.84)	0.64 (0.43; 0.94)	1.03 (0.59; 1.80)	0.46 (0.24; 0.88)	0.47 (0.27; 0.80)	1.39 (0.84; 2.30)	0.63 (0.29; 1.40)	1.12 (0.63; 1.98)	0.85 (0.39; 1.85)	1.00 (0.73; 1.37)	0.38 (0.19; 0.76)	Propranolol	0.93 (0.47; 1.82)	.	1.70 (1.12; 2.58)	.	.	0.82 (0.51; 1.29)	.	.
16	1.02 (0.36; 2.87)	0.67 (0.31; 1.46)	1.17 (0.33; 4.16)	1.00 (0.42; 2.37)	0.59 (0.27; 1.28)	0.96 (0.40; 2.29)	0.42 (0.17; 1.08)	0.43 (0.18; 1.03)	1.29 (0.56; 2.99)	0.59 (0.21; 1.67)	1.04 (0.43; 2.52)	0.79 (0.28; 2.21)	0.93 (0.44; 1.95)	0.35 (0.13; 0.93)	0.93 (0.47; 1.82)	Propranolol + Cinnarizine	.	.	.	.	.	.	.
17	2.21 (0.96; 5.10)	1.46 (0.87; 2.44)	2.51 (0.82; 7.70)	2.16 (1.01; 4.60)	1.28 (0.74; 2.19)	2.07 (1.09; 3.92)	0.91 (0.45; 1.87)	0.94 (0.51; 1.72)	2.78 (1.51; 5.14)	1.27 (0.49; 3.31)	2.25 (1.18; 4.27)	1.71 (0.74; 3.91)	2.01 (1.31; 3.08)	0.75 (0.32; 1.77)	2.01 (1.18; 3.41)	2.16 (0.92; 5.08)	Riboflavin	.	.	.	.	.	.
18	1.57 (0.73; 3.34)	1.03 (0.80; 1.34)	1.78 (0.62; 5.16)	1.53 (0.82; 2.85)	0.91 (0.63; 1.31)	1.47 (0.89; 2.43)	0.65 (0.35; 1.21)	0.67 (0.41; 1.09)	1.98 (1.29; 3.02)	0.90 (0.38; 2.12)	1.59 (0.93; 2.72)	1.21 (0.57; 2.56)	1.42 (1.12; 1.81)	0.54 (0.25; 1.13)	1.42 (1.04; 1.94)	1.53 (0.73; 3.21)	0.71 (0.43; 1.16)	Topiramate	1.59 (1.17; 2.17)	.	.	.	.
19	2.49 (1.10; 5.66)	1.64 (1.10; 2.46)	2.84 (0.94; 8.58)	2.44 (1.22; 4.89)	1.44 (0.89; 2.34)	2.33 (1.29; 4.22)	1.03 (0.52; 2.07)	1.06 (0.59; 1.90)	3.15 (1.86; 5.32)	1.44 (0.58; 3.56)	2.54 (1.37; 4.71)	1.93 (0.86; 4.34)	2.27 (1.53; 3.36)	0.85 (0.38; 1.91)	2.27 (1.46; 3.52)	2.44 (1.09; 5.44)	1.13 (0.63; 2.02)	1.59 (1.17; 2.17)	Topiramate + Vitamin D3	.	.	.	.
20	0.90 (0.39; 2.08)	0.59 (0.35; 0.99)	1.03 (0.34; 3.14)	0.88 (0.41; 1.87)	0.52 (0.30; 0.89)	0.84 (0.44; 1.60)	0.37 (0.18; 0.76)	0.38 (0.21; 0.70)	1.14 (0.62; 2.09)	0.52 (0.20; 1.35)	0.92 (0.48; 1.74)	0.70 (0.30; 1.59)	0.82 (0.53; 1.25)	0.31 (0.13; 0.72)	0.82 (0.48; 1.39)	0.88 (0.37; 2.07)	0.41 (0.22; 0.75)	0.57 (0.35; 0.94)	0.36 (0.20; 0.64)	Trazodone	.	.	.

© 2024 Kohandel Gargari O et al. *JAMA Network Open.*

21	1.16 (0.51; 2.61)	0.76 (0.49; 1.20)	1.32 (0.44; 3.97)	1.13 (0.60; 2.16)	0.67 (0.44; 1.02)	1.08 (0.59; 1.98)	0.48 (0.24; 0.95)	0.49 (0.28; 0.88)	1.46 (0.84; 2.56)	0.67 (0.28; 1.59)	1.18 (0.64; 2.17)	0.90 (0.40; 2.01)	1.05 (0.72; 1.54)	0.40 (0.20; 0.77)	1.05 (0.74; 1.50)	1.13 (0.53; 2.42)	0.52 (0.30; 0.93)	0.74 (0.49; 1.11)	0.46 (0.28; 0.77)	1.29 (0.73; 2.28)	Valproate	1.08 (0.44; 2.64)	.
22	1.25 (0.38; 4.19)	0.83 (0.30; 2.25)	1.43 (0.35; 5.89)	1.23 (0.41; 3.68)	0.73 (0.27; 1.94)	1.17 (0.40; 3.44)	0.52 (0.17; 1.60)	0.53 (0.18; 1.54)	1.58 (0.55; 4.53)	0.72 (0.21; 2.51)	1.28 (0.43; 3.76)	0.97 (0.29; 3.23)	1.14 (0.43; 3.00)	0.43 (0.14; 1.31)	1.14 (0.44; 2.97)	1.23 (0.38; 3.95)	0.57 (0.20; 1.64)	0.80 (0.30; 2.13)	0.50 (0.18; 1.40)	1.39 (0.48; 4.02)	1.08 (0.44; 2.64)	Valproate + Omega-3	.
23	1.29 (0.56; 2.99)	0.85 (0.50; 1.44)	1.47 (0.48; 4.51)	1.26 (0.59; 2.70)	0.75 (0.43; 1.29)	1.21 (0.63; 2.31)	0.53 (0.26; 1.10)	0.55 (0.30; 1.01)	1.63 (0.88; 3.02)	0.74 (0.28; 1.94)	1.31 (0.69; 2.51)	1.00 (0.43; 2.30)	1.17 (0.76; 1.82)	0.44 (0.19; 1.04)	1.17 (0.68; 2.01)	1.26 (0.53; 2.98)	0.58 (0.32; 1.08)	0.82 (0.50; 1.36)	0.52 (0.29; 0.93)	1.43 (0.78; 2.64)	1.11 (0.62; 1.99)	1.03 (0.36; 2.98)	Vitamin B Complex

© 2024 Kohandel Gargari O et al. *JAMA Network Open.*

eFigure 5. Network Splitting Analysis of Headache Frequency

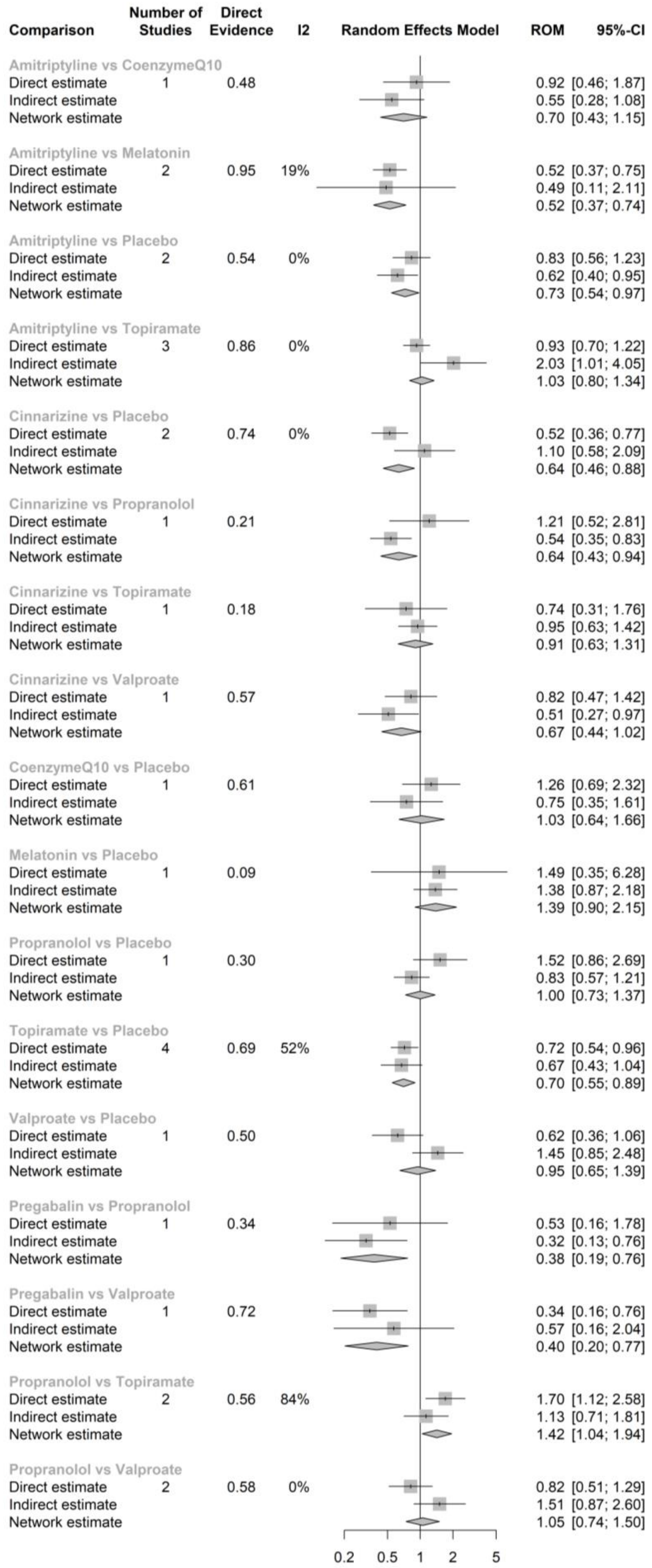

This figure presents the network splitting analysis of headache frequency, which examines the consistency between direct and indirect evidence within the network meta-analysis. By splitting the network into these two types of evidence, the analysis evaluates whether the conclusions drawn from the meta-analysis are consistent regardless of the source of evidence, ensuring the robustness and reliability of the overall results.

© 2024 Kohandel Gargari O et al. *JAMA Network Open.*

eFigure 6. Network Meta-Analysis Heatmap for Headache Frequency

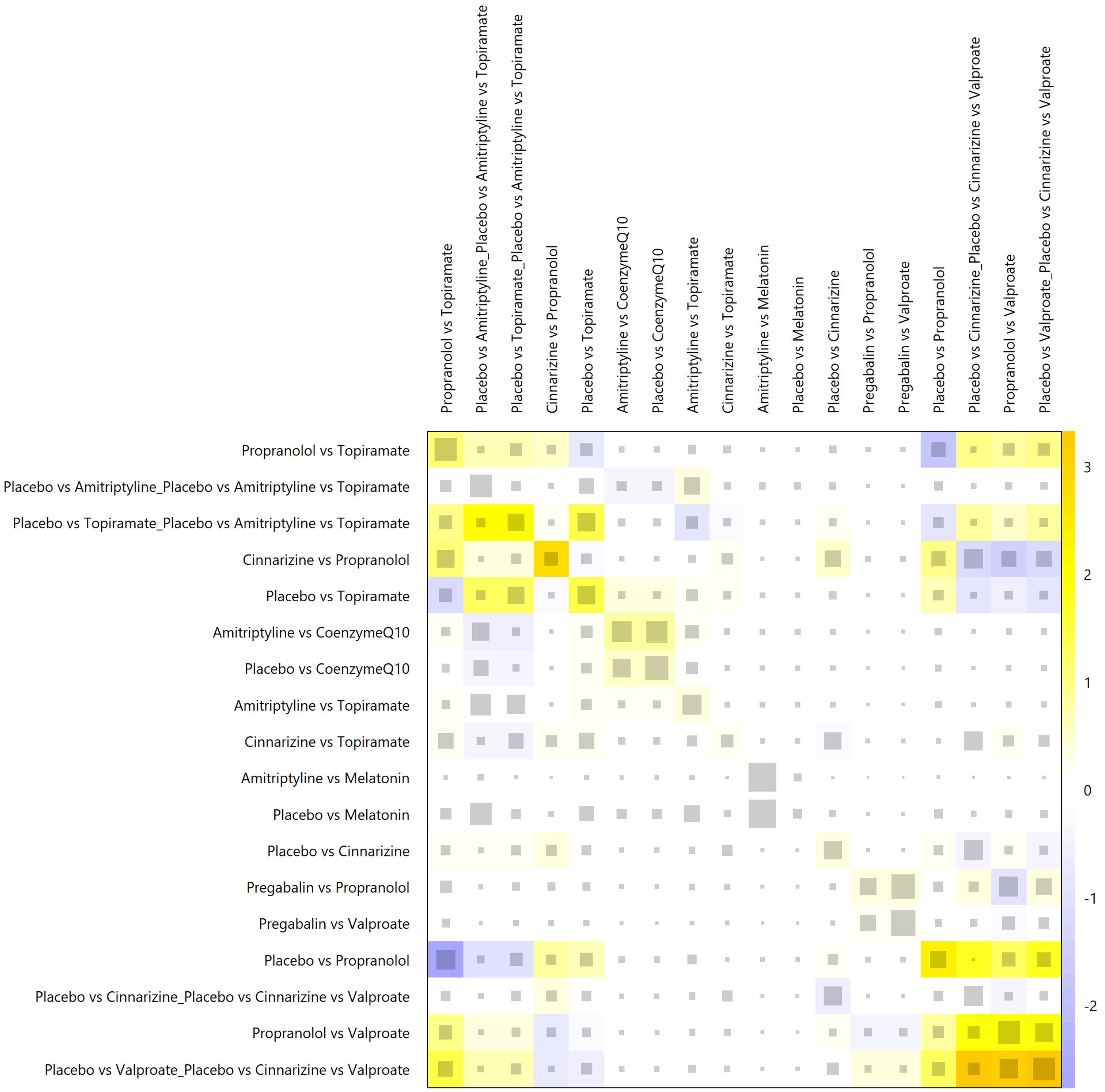

The net heat plot is a matrix visualization that highlights areas of inconsistency within the network meta-analysis. Each gray square's area represents the contribution of the direct estimate from the design in the column to the network estimate in the row. The colors indicate the change in inconsistency when relaxing the consistency assumption for single designs: cool colors (e.g., blue) indicate an increase in inconsistency, while warm colors (e.g., red) indicate a decrease. Diagonal colors show the inconsistency contribution of the corresponding design, whereas off-diagonal colors reflect the change in inconsistency between direct and indirect evidence. Clustering identifies hot spots of inconsistency, helping to locate potential sources for further investigation. Designs involving three or more treatments are marked with an underscore following the treatments of the design.

© 2024 Kohandel Gargari O et al. *JAMA Network Open.*

eFigure 7. Funnel Plot for 50% Headache Frequency Reduction Rate

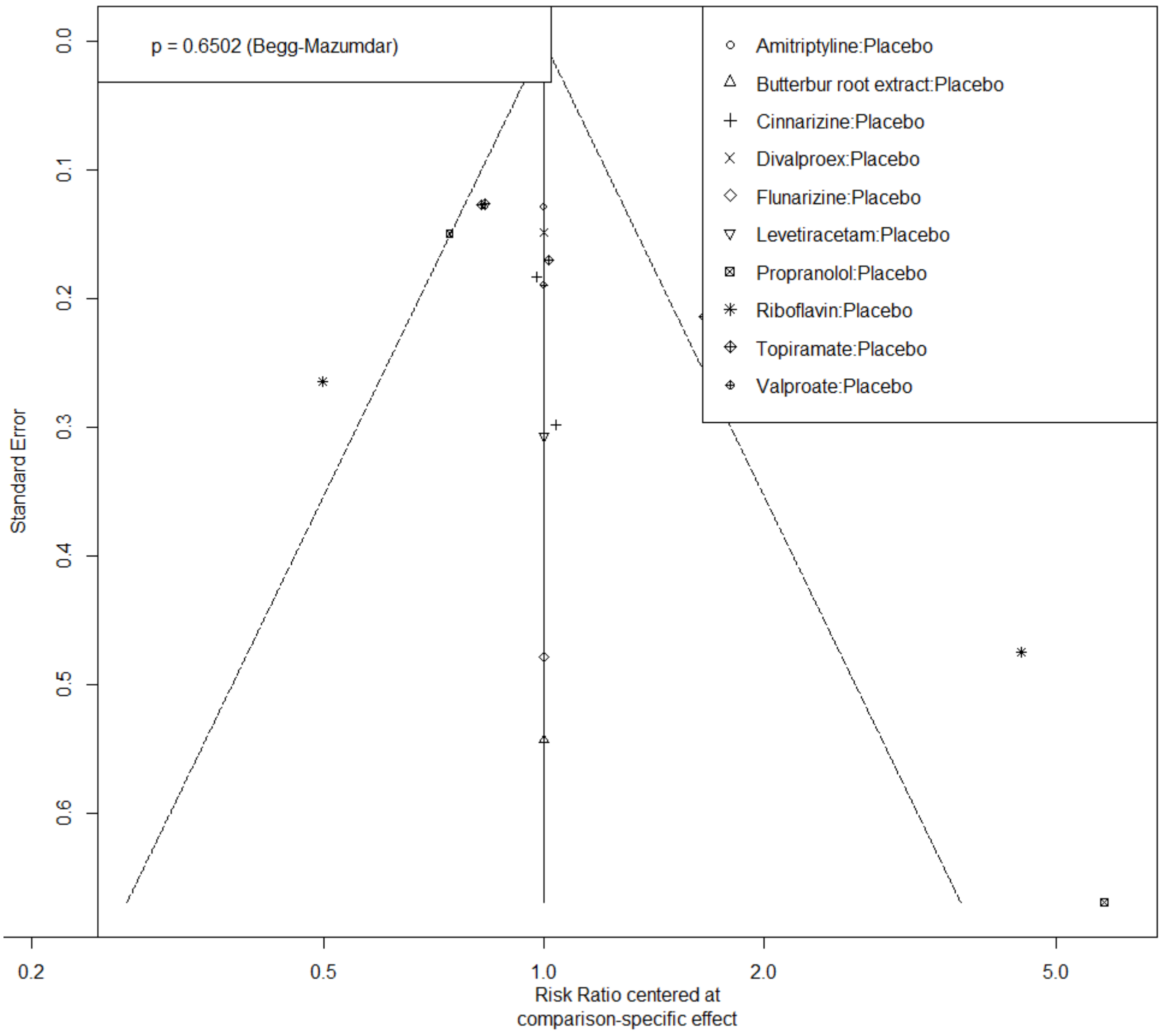

© 2024 Kohandel Gargari O et al. *JAMA Network Open.*

eTable 4. Network League for 50% Responder Rate

This table presents the network league table for the 50% headache reduction rate, comparing the relative efficacy of different interventions. The table displays the risk ratio for the 50% reduction rate between each pair of interventions, with corresponding 95% confidence intervals (CIs).

1	Amitriptyline	.	.	.	.	.	1.00 (0.59; 1.69)	1.31 (0.88; 1.94)	0.86 (0.58; 1.28)	.	.	.	0.95 (0.67; 1.36)	.	.
2	1.11 (0.33; 3.74)	Butterbur root extract	.	.	.	.	.	.	1.00 (0.31; 3.23)	.	.	.	.	.	.
3	0.76 (0.49; 1.18)	0.69 (0.20; 2.33)	Cinnarizine	.	.	.	.	.	1.73 (1.08; 2.80)	.	1.01 (0.58; 1.74)	.	1.31 (0.70; 2.43)	.	1.09 (0.62; 1.90)
4	1.21 (0.62; 2.34)	1.09 (0.30; 4.03)	1.59 (0.82; 3.10)	Divalproex	.	.	.	.	0.92 (0.52; 1.63)	.	.	.	.	.	.
5	0.28 (0.09; 0.84)	0.25 (0.05; 1.22)	0.36 (0.12; 1.11)	0.23 (0.07; 0.76)	Flunarizine	0.46 (0.23; 0.93)	.	.	4.00 (1.38; 11.55)	.	.	.	.	.	.
6	0.13 (0.03; 0.47)	0.11 (0.02; 0.65)	0.17 (0.04; 0.62)	0.10 (0.03; 0.42)	0.46 (0.23; 0.93)	Flunarizine + ALA	.	.	.	.	.	.	.	.	.
7	0.79 (0.51; 1.24)	0.72 (0.20; 2.56)	1.05 (0.59; 1.86)	0.66 (0.31; 1.40)	2.87 (0.89; 9.22)	6.26 (1.60; 24.49)	Levetiracetam	.	2.33 (1.07; 5.08)	.	.	.	.	.	.
8	1.31 (0.88; 1.94)	1.18 (0.33; 4.26)	1.73 (0.96; 3.11)	1.08 (0.50; 2.34)	4.74 (1.46; 15.38)	10.33 (2.62; 40.78)	1.65 (0.91; 2.99)	Melatonin	.	.	.	.	.	.	.
9	1.11 (0.80; 1.53)	1.00 (0.31; 3.23)	1.46 (1.04; 2.05)	0.92 (0.52; 1.63)	4.00 (1.38; 11.55)	8.73 (2.44; 31.20)	1.39 (0.86; 2.27)	0.84 (0.51; 1.41)	Placebo	.	0.92 (0.54; 1.56)	0.75 (0.41; 1.35)	0.91 (0.68; 1.22)	.	0.64 (0.35; 1.19)
10	0.59 (0.33; 1.05)	0.53 (0.15; 1.91)	0.77 (0.46; 1.31)	0.49 (0.23; 1.05)	2.13 (0.66; 6.90)	4.64 (1.18; 18.30)	0.74 (0.37; 1.48)	0.45 (0.22; 0.90)	0.53 (0.32; 0.89)	Pregabalin	1.81 (0.98; 3.33)	.	.	.	1.33 (0.75; 2.36)
11	0.93 (0.61; 1.40)	0.84 (0.25; 2.82)	1.22 (0.87; 1.72)	0.77 (0.40; 1.48)	3.35 (1.11; 10.12)	7.31 (1.97; 27.14)	1.17 (0.67; 2.05)	0.71 (0.40; 1.26)	0.84 (0.61; 1.15)	1.58 (1.01; 2.46)	Propranolol	.	0.76 (0.43; 1.32)	.	1.11 (0.74; 1.66)
12	0.83 (0.42; 1.63)	0.75 (0.20; 2.78)	1.09 (0.55; 2.16)	0.68 (0.30; 1.56)	2.99 (0.89; 10.08)	6.53 (1.60; 26.61)	1.04 (0.48; 2.25)	0.63 (0.29; 1.38)	0.75 (0.41; 1.35)	1.41 (0.64; 3.08)	0.89 (0.46; 1.75)	Riboflavin	.	.	.
13	0.92 (0.67; 1.27)	0.83 (0.25; 2.76)	1.21 (0.86; 1.71)	0.76 (0.41; 1.42)	3.32 (1.12; 9.88)	7.25 (1.98; 26.55)	1.16 (0.70; 1.92)	0.70 (0.42; 1.17)	0.83 (0.65; 1.06)	1.56 (0.94; 2.60)	0.99 (0.73; 1.36)	1.11 (0.59; 2.11)	Topiramate	0.76 (0.49; 1.18)	0.95 (0.54; 1.67)
14	0.70 (0.40; 1.21)	0.63 (0.18; 2.26)	0.92 (0.52; 1.61)	0.58 (0.27; 1.24)	2.52 (0.78; 8.16)	5.50 (1.40; 21.66)	0.88 (0.45; 1.72)	0.53 (0.27; 1.05)	0.63 (0.38; 1.05)	1.19 (0.61; 2.32)	0.75 (0.44; 1.29)	0.84 (0.39; 1.84)	0.76 (0.49; 1.18)	Topiramate + VitD3	.
15	0.88 (0.57; 1.37)	0.80 (0.24; 2.72)	1.17 (0.81; 1.68)	0.73 (0.38; 1.43)	3.20 (1.05; 9.75)	6.98 (1.87; 26.11)	1.12 (0.63; 1.98)	0.68 (0.38; 1.21)	0.80 (0.57; 1.13)	1.51 (0.97; 2.33)	0.96 (0.71; 1.28)	1.07 (0.54; 2.12)	0.96 (0.69; 1.34)	1.27 (0.73; 2.21)	Valproate

© 2024 Kohandel Gargari O et al. *JAMA Network Open.*

eFigure 8. Net Splitting Analysis for 50% Headache Frequency Reduction Rate

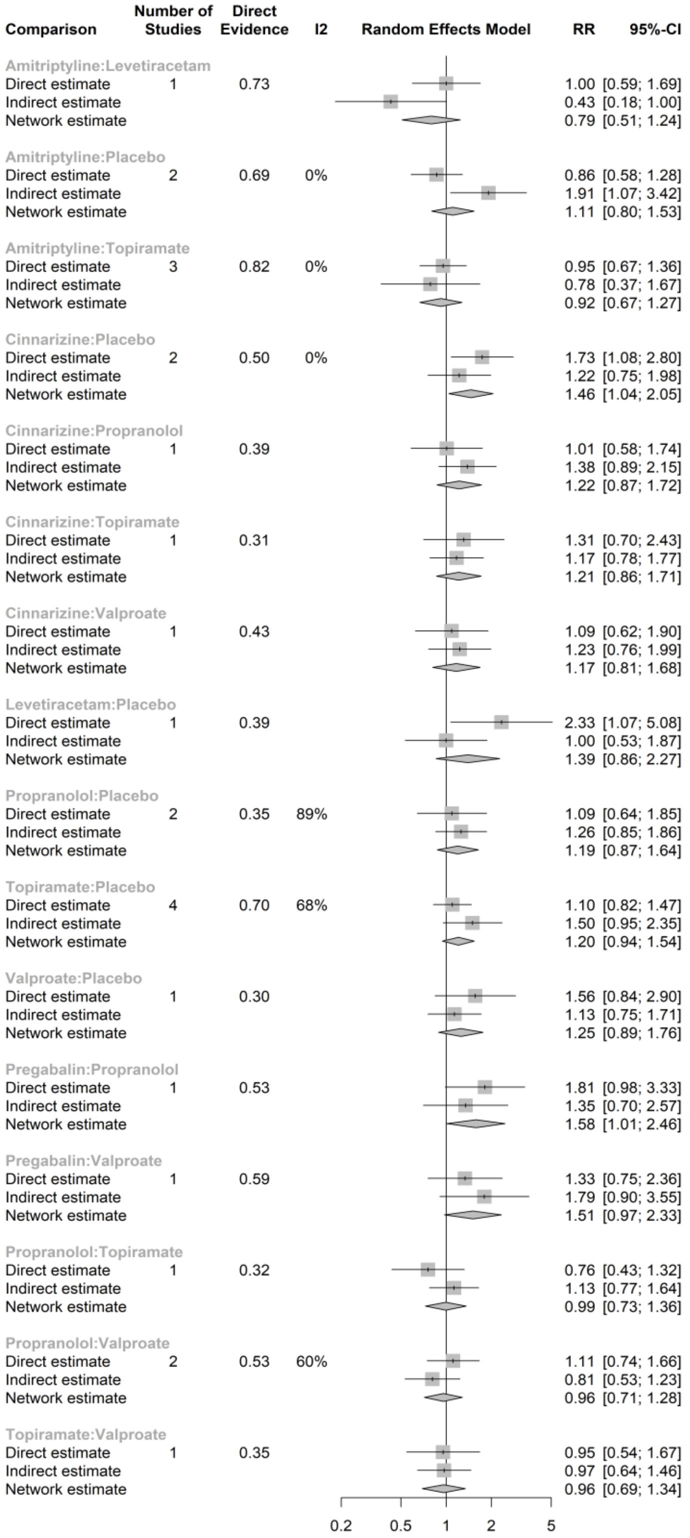

This figure presents the network splitting analysis for the 50% headache frequency reduction rate, which examines the consistency between direct and indirect evidence within the network meta-analysis. By splitting the network into these two types of evidence, the analysis evaluates whether the conclusions drawn from the meta-analysis are consistent regardless of the source of evidence, ensuring the robustness and reliability of the overall results.

© 2024 Kohandel Gargari O et al. *JAMA Network Open.*

eFigure 9. Network Meta-Analysis Heatmap for 50% Headache Frequency Reduction Rate

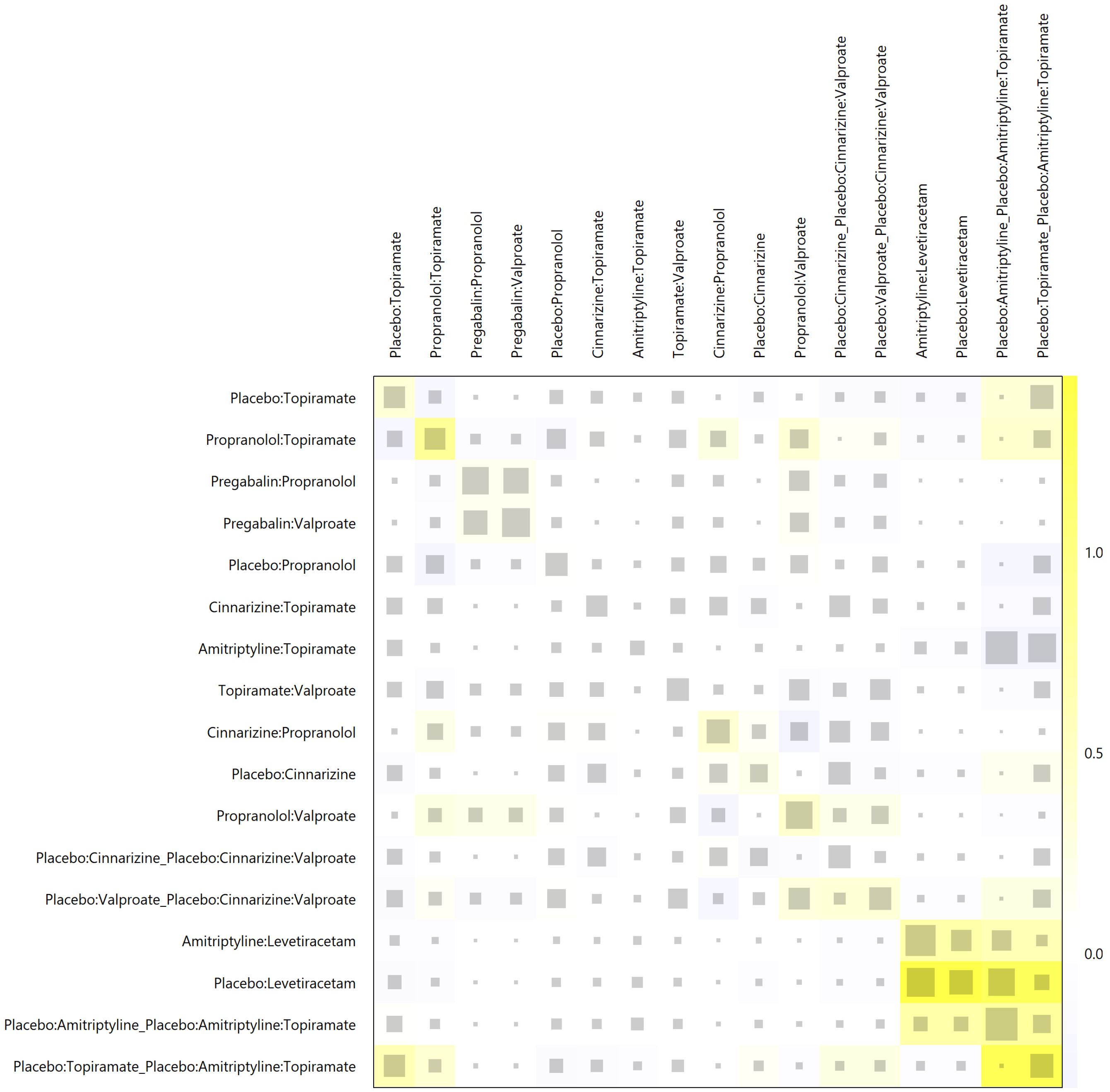

The net heat plot is a matrix visualization that highlights areas of inconsistency within the network meta-analysis. Each gray square's area represents the contribution of the direct estimate from the design in the column to the network estimate in the row. The colors indicate the change in inconsistency when relaxing the consistency assumption for single designs: cool colors (e.g., blue) indicate an increase in inconsistency, while warm colors (e.g., red) indicate a decrease. Diagonal colors show the inconsistency contribution of the corresponding design, whereas off-diagonal colors reflect the change in inconsistency between direct and indirect evidence. Clustering identifies hot spots of inconsistency, helping to locate potential sources for further investigation. Designs involving three or more treatments are marked with an underscore following the treatments of the design.

© 2024 Kohandel Gargari O et al. *JAMA Network Open.*

eFigure 10. Funnel Plot for Migraine Intensity

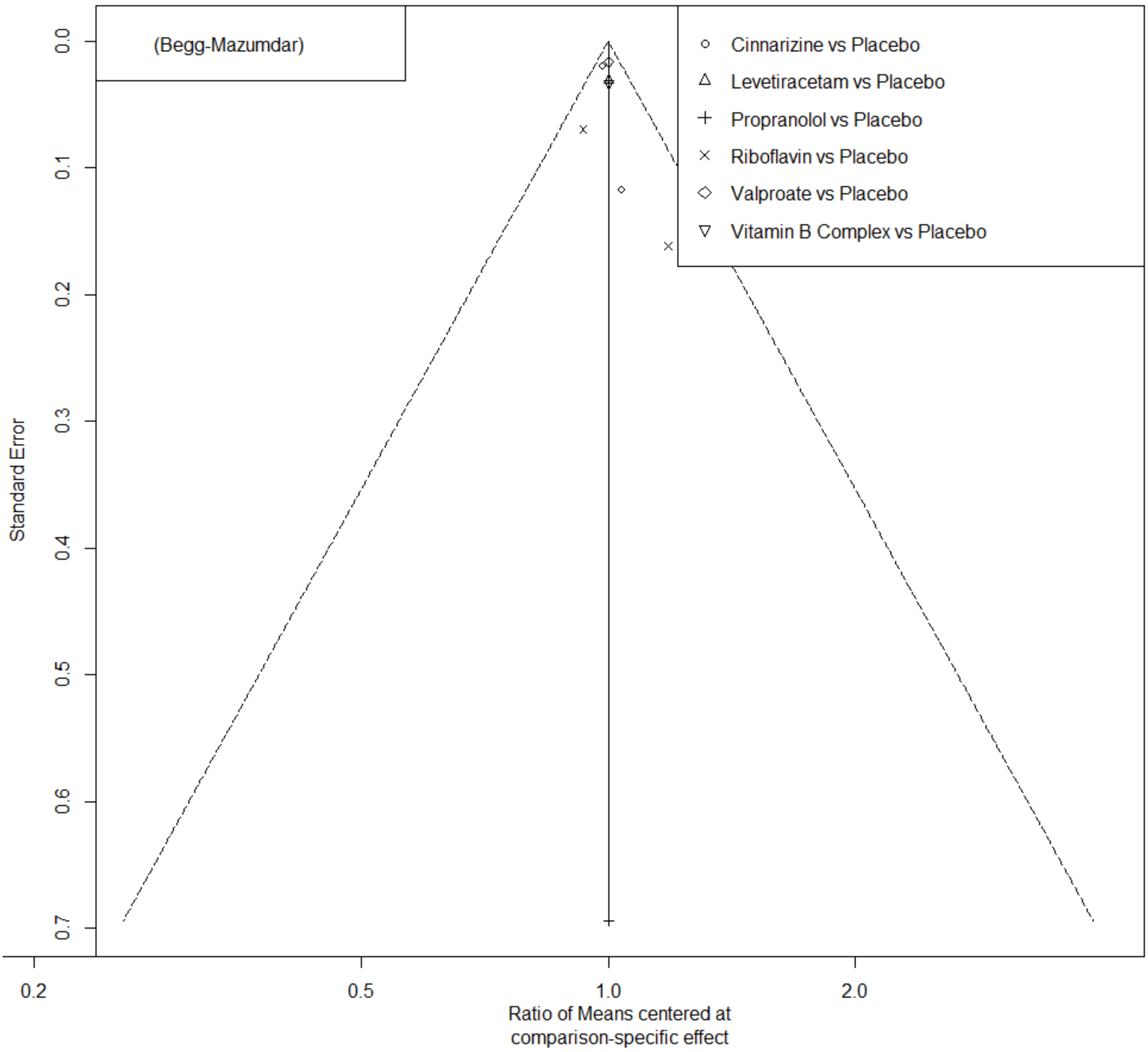

© 2024 Kohandel Gargari O et al. *JAMA Network Open.*

eFigure 11. Network Splitting Analysis For Headache Intensity

Comparison	Number of Studies	Direct Evidence	I2	ROM	95%-CI
**Cinnarizine vs Placebo**					
Direct estimate	2	0.98	0%	0.64	[0.54; 0.76]
Indirect estimate				0.52	[0.16; 1.65]
Network estimate				0.64	[0.54; 0.76]
**Cinnarizine vs Topiramate**					
Direct estimate	1	0.35		0.74	[0.42; 1.31]
Indirect estimate				0.95	[0.63; 1.44]
Network estimate				0.87	[0.63; 1.22]
**Cinnarizine vs Valproate**					
Direct estimate	1	0.85		1.09	[0.89; 1.34]
Indirect estimate				0.98	[0.60; 1.59]
Network estimate				1.07	[0.89; 1.30]
**Propranolol vs Placebo**					
Direct estimate	1	0.08		1.26	[0.32; 4.99]
Indirect estimate				0.83	[0.55; 1.25]
Network estimate				0.86	[0.58; 1.27]
**Valproate vs Placebo**					
Direct estimate	1	0.88		0.58	[0.47; 0.71]
Indirect estimate				0.74	[0.42; 1.31]
Network estimate				0.60	[0.49; 0.72]
**Propranolol vs Topiramate**					
Direct estimate	2	0.98	88%	1.17	[0.95; 1.43]
Indirect estimate				1.77	[0.43; 7.32]
Network estimate				1.18	[0.96; 1.44]
**Topiramate vs Valproate**					
Direct estimate	1	0.68		1.11	[0.76; 1.62]
Indirect estimate				1.53	[0.88; 2.67]
Network estimate				1.23	[0.90; 1.68]

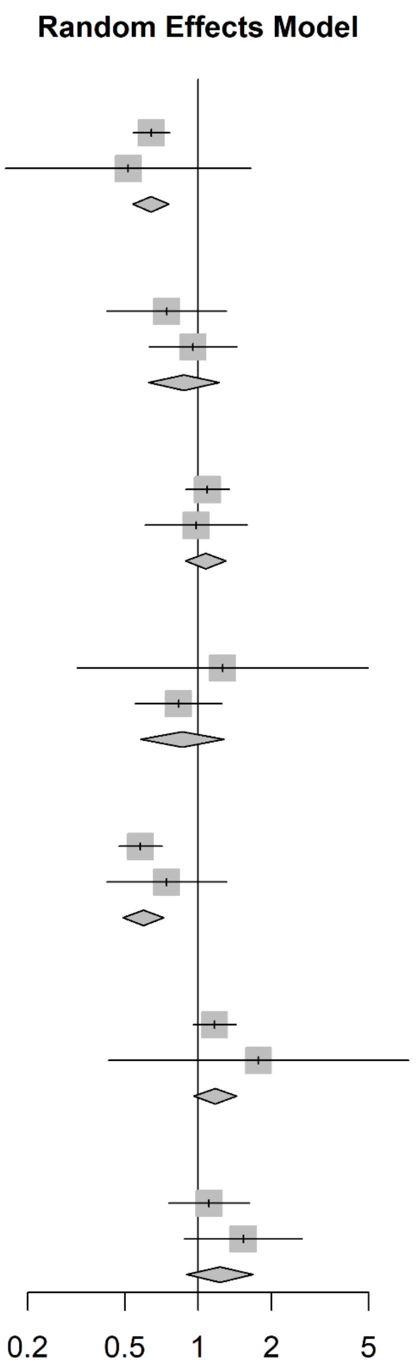

This figure presents the network splitting analysis for headache intensity, which examines the consistency between direct and indirect evidence within the network meta-analysis. By splitting the network into these two types of evidence, the analysis evaluates whether the conclusions drawn from the meta-analysis are consistent regardless of the source of evidence, ensuring the robustness and reliability of the overall results.

© 2024 Kohandel Gargari O et al. *JAMA Network Open.*

eTable 5. Network League Table for Headache Intensity

This table presents the network league table for headache intensity, comparing the relative efficacy of different interventions. The table displays the ratio of means for headache intensity between each pair of interventions, with corresponding 95% confidence intervals (CIs).

1	Amitriptyline	.	0.84 (0.56; 1.25)	.	0.60 (0.49; 0.73)	.	.	.	.	.	1.08 (0.88; 1.33)	.	.	.
2	1.24 (0.84; 1.83)	Cinnarizine	.	.	.	0.64 (0.54; 0.76)	.	.	.	.	0.74 (0.42; 1.31)	.	1.09 (0.89; 1.34)	.
3	0.84 (0.56; 1.25)	0.68 (0.39; 1.18)	CoenzymeQ10	.	.	.	.	.	.	.	.	.	.	.
4	1.27 (0.81; 2.00)	1.02 (0.78; 1.34)	1.51 (0.83; 2.77)	Levetiracetam	.	0.62 (0.50; 0.77)	.	.	.	.	.	.	.	.
5	0.60 (0.49; 0.73)	0.48 (0.31; 0.75)	0.71 (0.45; 1.11)	0.47 (0.29; 0.77)	Melatonin	.	.	.	.	.	.	.	.	.
6	0.79 (0.53; 1.18)	0.64 (0.54; 0.76)	0.94 (0.54; 1.66)	0.62 (0.50; 0.77)	1.33 (0.85; 2.08)	Placebo	.	0.79 (0.20; 3.14)	.	0.92 (0.75; 1.12)	.	.	1.73 (1.41; 2.12)	1.23 (0.99; 1.52)
7	1.40 (0.75; 2.60)	1.13 (0.67; 1.92)	1.67 (0.80; 3.49)	1.10 (0.62; 1.95)	2.35 (1.22; 4.51)	1.77 (1.04; 3.01)	Pregabalin	.	.	.	.	.	0.95 (0.58; 1.56)	.
8	0.92 (0.69; 1.23)	0.74 (0.51; 1.09)	1.10 (0.67; 1.79)	0.73 (0.46; 1.13)	1.54 (1.08; 2.20)	1.16 (0.78; 1.72)	0.66 (0.35; 1.22)	Propranolol	1.91 (1.47; 2.49)	.	1.17 (0.95; 1.43)	.	.	.
9	1.76 (1.19; 2.60)	1.42 (0.89; 2.26)	2.10 (1.20; 3.66)	1.39 (0.83; 2.33)	2.95 (1.90; 4.58)	2.22 (1.39; 3.56)	1.26 (0.64; 2.45)	1.91 (1.47; 2.49)	Propranolol + Cinnarizine	.	.	.	.	.
10	0.73 (0.46; 1.14)	0.59 (0.45; 0.76)	0.86 (0.47; 1.58)	0.57 (0.43; 0.77)	1.22 (0.74; 1.99)	0.92 (0.75; 1.12)	0.52 (0.29; 0.91)	0.79 (0.51; 1.23)	0.41 (0.25; 0.69)	Riboflavin	.	.	.	.
11	1.08 (0.88; 1.33)	0.87 (0.63; 1.22)	1.29 (0.82; 2.02)	0.85 (0.57; 1.28)	1.82 (1.36; 2.42)	1.37 (0.97; 1.93)	0.77 (0.43; 1.39)	1.18 (0.96; 1.44)	0.61 (0.44; 0.86)	1.49 (1.00; 2.23)	Topiramate	1.03 (0.83; 1.26)	1.11 (0.76; 1.62)	.
12	1.11 (0.83; 1.49)	0.90 (0.60; 1.33)	1.32 (0.81; 2.17)	0.87 (0.55; 1.38)	1.86 (1.30; 2.66)	1.40 (0.94; 2.10)	0.79 (0.43; 1.48)	1.21 (0.90; 1.61)	0.63 (0.43; 0.93)	1.53 (0.97; 2.41)	1.03 (0.83; 1.26)	Topiramate + Vit-D3	.	.
13	1.33 (0.92; 1.93)	1.07 (0.89; 1.30)	1.58 (0.92; 2.74)	1.05 (0.79; 1.40)	2.23 (1.46; 3.41)	1.68 (1.38; 2.04)	0.95 (0.58; 1.56)	1.44 (1.00; 2.09)	0.76 (0.48; 1.19)	1.83 (1.38; 2.43)	1.23 (0.90; 1.68)	1.20 (0.82; 1.75)	Valproate	.
14	0.97 (0.62; 1.53)	0.79 (0.60; 1.03)	1.16 (0.63; 2.12)	0.77 (0.57; 1.04)	1.63 (0.99; 2.69)	1.23 (0.99; 1.52)	0.70 (0.39; 1.23)	1.06 (0.68; 1.66)	0.55 (0.33; 0.93)	1.34 (1.00; 1.81)	0.90 (0.60; 1.35)	0.88 (0.56; 1.38)	0.73 (0.55; 0.98)	Vitamin B Complex

© 2024 Kohandel Gargari O et al. *JAMA Network Open.*

eFigure 12. Network Meta-Analysis Heatmap For Headache Intensity

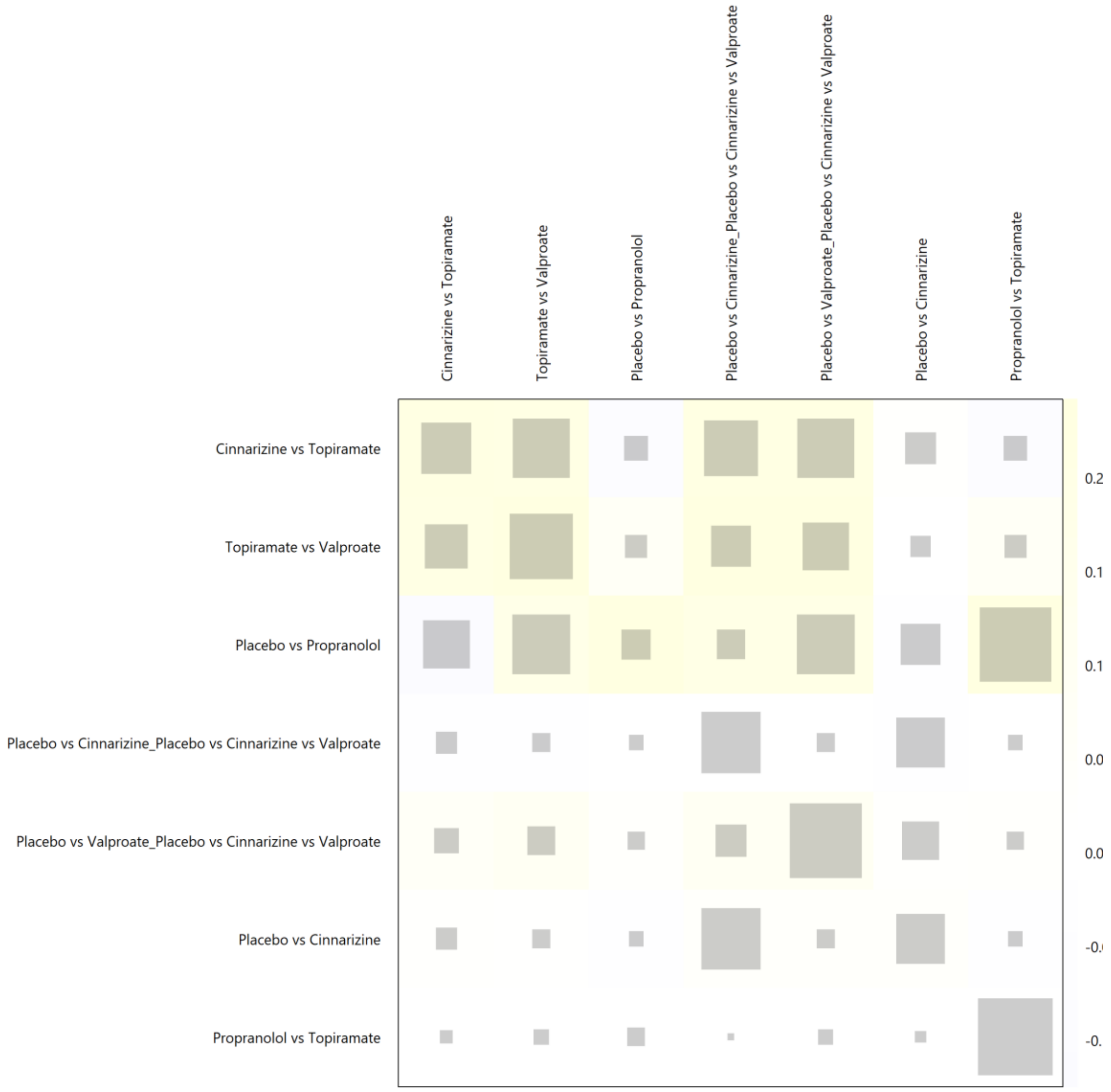

The net heat plot is a matrix visualization that highlights areas of inconsistency within the network meta-analysis. Each gray square's area represents the contribution of the direct estimate from the design in the column to the network estimate in the row. The colors indicate the change in inconsistency when relaxing the consistency assumption for single designs: cool colors (e.g., blue) indicate an increase in inconsistency, while warm colors (e.g., red) indicate a decrease. Diagonal colors show the inconsistency contribution of the corresponding design, whereas off-diagonal colors reflect the change in inconsistency between direct and indirect evidence. Clustering identifies hot spots of inconsistency, helping to locate potential sources for further investigation. Designs involving three or more treatments are marked with an underscore following the treatments of the design.

© 2024 Kohandel Gargari O et al. *JAMA Network Open.*

eFigure 13. Funnel Plot for Quality-of-Life Outcome

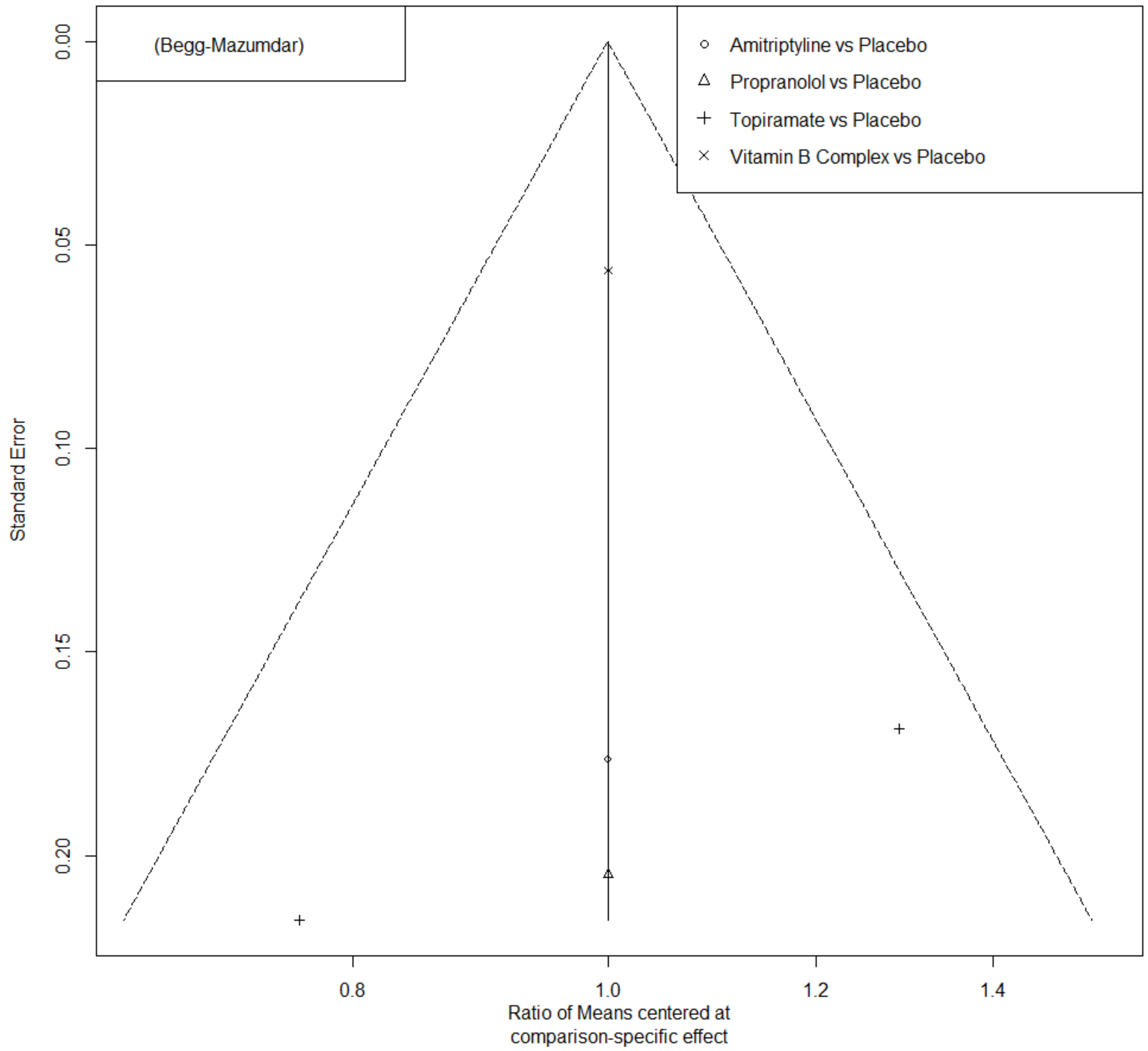

© 2024 Kohandel Gargari O et al. *JAMA Network Open.*

eFigure 14. Net Splitting Analysis for Quality of Life

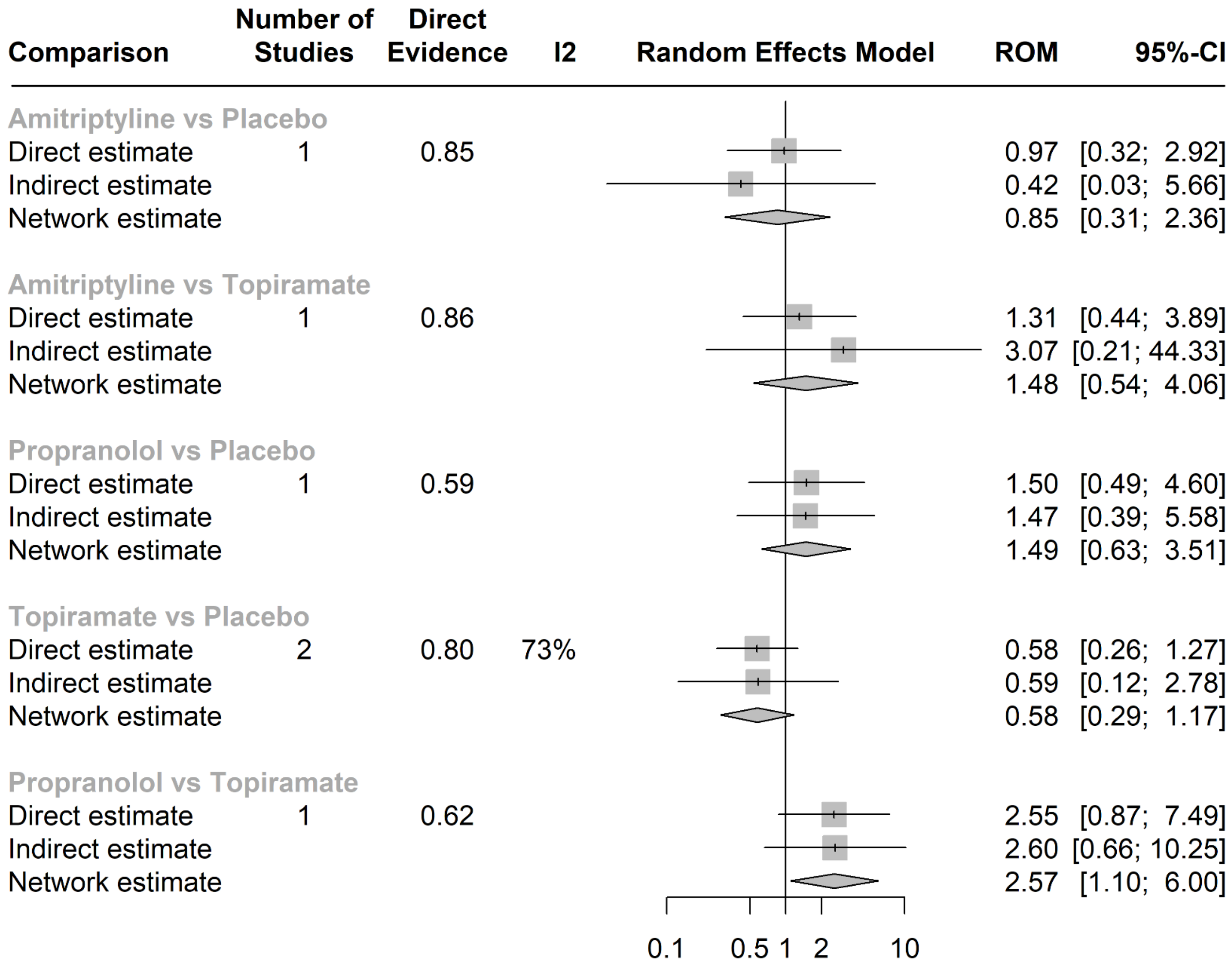

This figure presents the network splitting analysis for quality of life, which examines the consistency between direct and indirect evidence within the network meta-analysis. By splitting the network into these two types of evidence, the analysis evaluates whether the conclusions drawn from the meta-analysis are consistent regardless of the source of evidence, ensuring the robustness and reliability of the overall results.

© 2024 Kohandel Gargari O et al. *JAMA Network Open.*

eFigure 15. Network Meta-Analysis Heatmap for Quality of Life

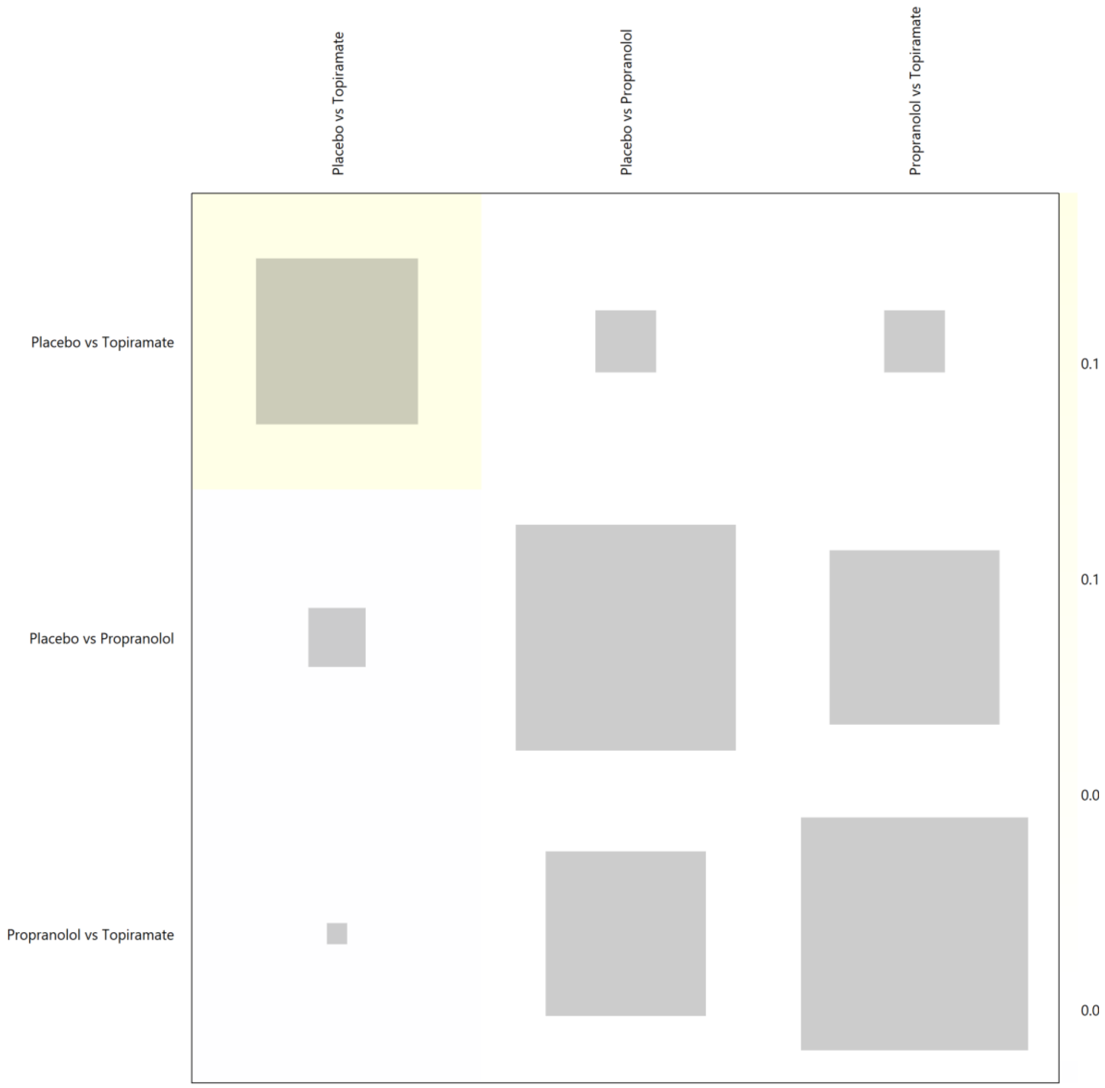

The net heat plot is a matrix visualization that highlights areas of inconsistency within the network meta-analysis. Each gray square's area represents the contribution of the direct estimate from the design in the column to the network estimate in the row. The colors indicate the change in inconsistency when relaxing the consistency assumption for single designs: cool colors (e.g., blue) indicate an increase in inconsistency, while warm colors (e.g., red) indicate a decrease. Diagonal colors show the inconsistency contribution of the corresponding design, whereas off-diagonal colors reflect the change in inconsistency between direct and indirect evidence. Clustering identifies hot spots of inconsistency, helping to locate potential sources for further investigation. Designs involving three or more treatments are marked with an underscore following the treatments of the design.

© 2024 Kohandel Gargari O et al. *JAMA Network Open.*

eTable 6. Network League Table for Quality of Life

This table presents the network league table for quality of life, comparing the relative efficacy of different interventions. The table displays the ratio of means for quality of life, measured by the Pediatric Migraine Disability Assessment (PedMIDAS) tool, between each pair of interventions, with corresponding 95% confidence intervals (CIs).

1	Amitriptyline	.	1.79 (0.53; 6.06)	0.62 (0.29; 1.32)	0.97 (0.32; 2.92)	.	1.31 (0.44; 3.89)	.	.	.	.
2	0.16 (0.02; 1.17)	Carnitine	.	.	.	3.51 (0.75; 16.38)	.	.	.	.	.
3	1.79 (0.53; 6.06)	10.95 (1.08; 111.07)	CoenzymeQ10	.	.	.	.	.	.	.	.
4	0.62 (0.29; 1.32)	3.81 (0.46; 31.33)	0.35 (0.08; 1.46)	Melatonin	.	.	.	.	.	.	.
5	0.85 (0.31; 2.36)	5.22 (0.89; 30.42)	0.48 (0.10; 2.33)	1.37 (0.39; 4.85)	Placebo	0.67 (0.22; 2.05)	1.74 (0.79; 3.82)	.	.	.	1.19 (0.41; 3.41)
6	0.57 (0.17; 1.96)	3.51 (0.75; 16.38)	0.32 (0.06; 1.81)	0.92 (0.22; 3.89)	0.67 (0.29; 1.59)	Propranolol	2.55 (0.87; 7.49)	.	.	.	.
7	1.48 (0.54; 4.06)	9.03 (1.56; 52.36)	0.82 (0.17; 4.02)	2.37 (0.67; 8.36)	1.73 (0.86; 3.50)	2.57 (1.10; 6.00)	Topiramate	1.28 (0.61; 2.72)	1.07 (0.37; 3.09)	.	.
8	1.90 (0.54; 6.68)	11.59 (1.71; 78.41)	1.06 (0.18; 6.12)	3.05 (0.70; 13.20)	2.22 (0.79; 6.22)	3.30 (1.06; 10.24)	1.28 (0.61; 2.72)	Topiramate + Vit-D3	.	.	.
9	1.58 (0.37; 6.85)	9.68 (1.24; 75.37)	0.88 (0.13; 5.95)	2.54 (0.49; 13.18)	1.86 (0.52; 6.62)	2.76 (0.71; 10.70)	1.07 (0.37; 3.09)	0.84 (0.23; 3.06)	Valproate	0.93 (0.32; 2.67)	.
10	1.47 (0.24; 8.93)	8.97 (0.89; 90.26)	0.82 (0.09; 7.25)	2.36 (0.33; 16.67)	1.72 (0.33; 8.99)	2.55 (0.46; 14.27)	0.99 (0.22; 4.44)	0.77 (0.14; 4.13)	0.93 (0.32; 2.67)	Valproate + Omega-3	.
11	1.01 (0.23; 4.38)	6.19 (0.79; 48.31)	0.57 (0.08; 3.80)	1.63 (0.31; 8.43)	1.19 (0.41; 3.41)	1.76 (0.45; 6.87)	0.69 (0.19; 2.43)	0.53 (0.12; 2.33)	0.64 (0.12; 3.33)	0.69 (0.10; 4.91)	Vitamin B Complex

© 2024 Kohandel Gargari O et al. *JAMA Network Open.*

eFigure 16. Funnel Plot for Headache Duration

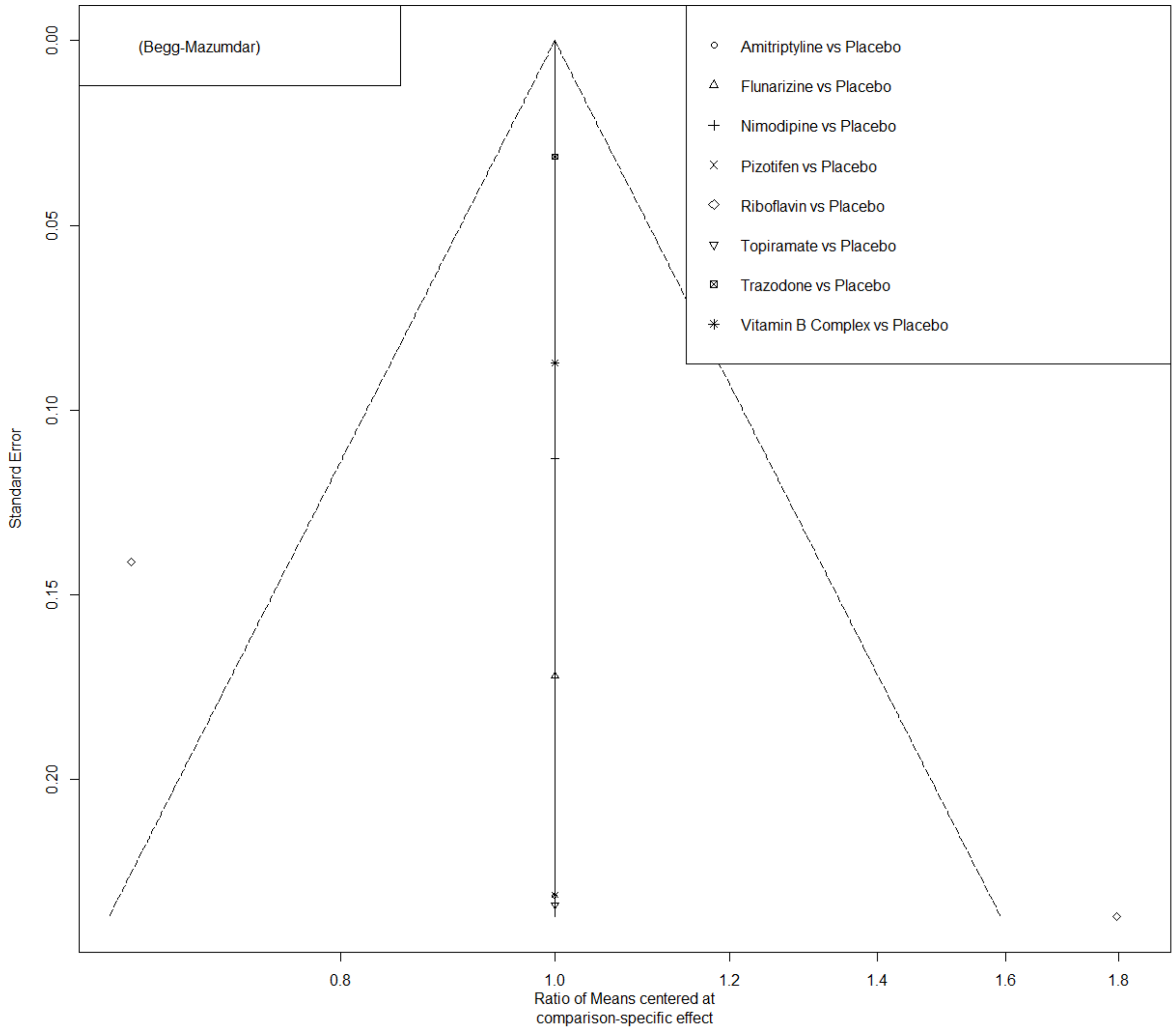

© 2024 Kohandel Gargari O et al. *JAMA Network Open.*

eFigure 17. Network Splitting Analysis for Headache Duration

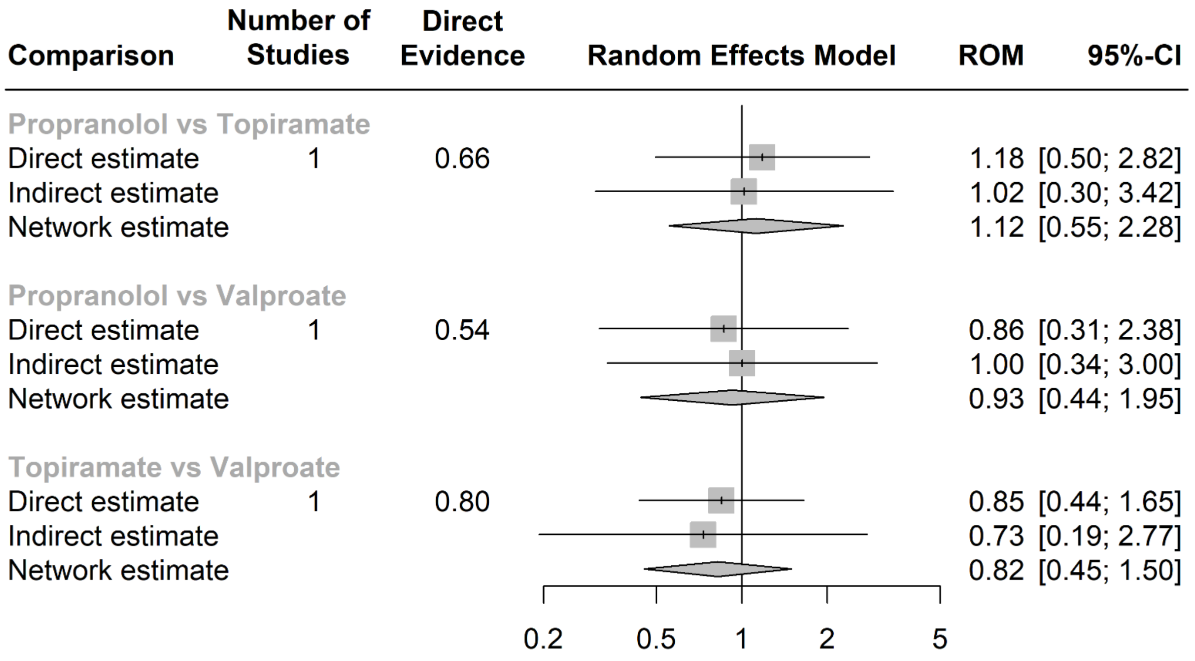

This figure presents the network splitting analysis for headache duration, which examines the consistency between direct and indirect evidence within the network meta-analysis. By splitting the network into these two types of evidence, the analysis evaluates whether the conclusions drawn from the meta-analysis are consistent regardless of the source of evidence, ensuring the robustness and reliability of the overall results.

© 2024 Kohandel Gargari O et al. *JAMA Network Open.*

eTable 7. Network League Table for Headache Duration
This table presents the network league table for headache duration, comparing the relative efficacy of different interventions. The table displays the ratio of means for headache duration between each pair of interventions, with corresponding 95% confidence intervals (CIs).

1	Amitriptyline	0.86 (0.18; 4.09)	.	0.58 (0.30; 1.15)	.	.	0.76 (0.36; 1.60)	.	.	.	.	0.90 (0.44; 1.85)	.	.	.	.
2	0.86 (0.18; 4.09)	CoenzymeQ10	.	.	.	.	.	.	.	.	.	.	.	.	.	.
3	0.95 (0.34; 2.60)	1.09 (0.17; 6.99)	Flunarizine	.	.	.	0.80 (0.40; 1.58)	.	.	.	.	.	.	.	.	.
4	0.58 (0.30; 1.15)	0.67 (0.12; 3.68)	0.62 (0.18; 2.08)	Melatonin	.	.	.	.	.	.	.	.	.	.	.	.
5	0.67 (0.25; 1.78)	0.78 (0.12; 4.87)	0.71 (0.28; 1.80)	1.15 (0.35; 3.78)	Nimodipine	.	1.13 (0.60; 2.13)	.	.	.	.	.	.	.	.	.
6	0.79 (0.27; 2.27)	0.91 (0.14; 5.99)	0.84 (0.30; 2.30)	1.36 (0.39; 4.75)	1.18 (0.44; 3.14)	Pizotifen	0.96 (0.45; 2.02)	.	.	.	.	.	.	.	.	.
7	0.76 (0.36; 1.60)	0.88 (0.16; 4.92)	0.80 (0.40; 1.58)	1.30 (0.47; 3.56)	1.13 (0.60; 2.13)	0.96 (0.45; 2.02)	Placebo	.	.	.	1.25 (0.76; 2.05)	1.19 (0.56; 2.53)	.	0.63 (0.35; 1.15)	.	1.14 (0.61; 2.11)
8	0.76 (0.20; 2.93)	0.88 (0.11; 6.90)	0.81 (0.18; 3.71)	1.31 (0.29; 5.91)	1.14 (0.25; 5.12)	0.97 (0.20; 4.57)	1.01 (0.26; 3.94)	Pregabalin	.	.	.	.	.	.	0.97 (0.37; 2.57)	.
9	0.80 (0.29; 2.20)	0.93 (0.15; 5.93)	0.85 (0.25; 2.93)	1.38 (0.41; 4.64)	1.20 (0.36; 4.02)	1.02 (0.29; 3.63)	1.06 (0.38; 2.97)	1.05 (0.31; 3.57)	Propranolol	1.23 (0.58; 2.62)	.	1.18 (0.50; 2.82)	.	.	0.86 (0.31; 2.38)	.
10	0.99 (0.28; 3.48)	1.15 (0.16; 8.46)	1.05 (0.25; 4.45)	1.70 (0.41; 7.08)	1.48 (0.36; 6.14)	1.25 (0.29; 5.50)	1.31 (0.37; 4.69)	1.30 (0.31; 5.45)	1.23 (0.58; 2.62)	Propranolol + Cinnarizine	.	.	.	.	.	.
11	0.94 (0.39; 2.31)	1.09 (0.18; 6.58)	1.00 (0.43; 2.32)	1.62 (0.53; 4.99)	1.41 (0.63; 3.15)	1.20 (0.49; 2.93)	1.25 (0.76; 2.05)	1.24 (0.29; 5.27)	1.18 (0.37; 3.68)	0.95 (0.24; 3.75)	Riboflavin	.	.	.	.	.
12	0.90 (0.44; 1.85)	1.05 (0.19; 5.79)	0.96 (0.35; 2.63)	1.55 (0.58; 4.16)	1.35 (0.51; 3.60)	1.14 (0.40; 3.29)	1.19 (0.56; 2.53)	1.18 (0.38; 3.69)	1.12 (0.55; 2.28)	0.91 (0.32; 2.56)	0.96 (0.39; 2.35)	Topiramate	1.01 (0.65; 1.57)	.	0.85 (0.44; 1.65)	.
13	0.91 (0.39; 2.12)	1.05 (0.18; 6.18)	0.96 (0.32; 2.92)	1.56 (0.53; 4.61)	1.36 (0.46; 3.99)	1.15 (0.37; 3.64)	1.20 (0.50; 2.88)	1.19 (0.35; 4.05)	1.13 (0.49; 2.61)	0.92 (0.30; 2.83)	0.96 (0.35; 2.63)	1.01 (0.65; 1.57)	Topiramate + Vit-D3	.	.	.
14	0.48 (0.18; 1.25)	0.55 (0.09; 3.44)	0.51 (0.21; 1.26)	0.82 (0.25; 2.66)	0.72 (0.30; 1.71)	0.61 (0.23; 1.58)	0.63 (0.35; 1.15)	0.63 (0.14; 2.78)	0.60 (0.18; 1.96)	0.48 (0.12; 1.98)	0.51 (0.23; 1.10)	0.53 (0.20; 1.38)	0.53 (0.18; 1.51)	Trazodone	.	.
15	0.74 (0.29; 1.89)	0.86 (0.14; 5.27)	0.79 (0.24; 2.55)	1.28 (0.40; 4.04)	1.11 (0.35; 3.50)	0.94 (0.28; 3.17)	0.98 (0.38; 2.56)	0.97 (0.37; 2.57)	0.93 (0.44; 1.95)	0.75 (0.26; 2.16)	0.79 (0.27; 2.31)	0.82 (0.45; 1.50)	0.82 (0.39; 1.72)	1.55 (0.50; 4.80)	Valproate	.
16	0.86 (0.33; 2.27)	1.00 (0.16; 6.22)	0.91 (0.36; 2.28)	1.48 (0.45; 4.82)	1.28 (0.53; 3.11)	1.09 (0.41; 2.87)	1.14 (0.61; 2.11)	1.13 (0.25; 5.03)	1.07 (0.32; 3.55)	0.87 (0.21; 3.58)	0.91 (0.41; 2.01)	0.95 (0.36; 2.51)	0.94 (0.32; 2.75)	1.79 (0.76; 4.23)	1.16 (0.37; 3.61)	Vitamin B Complex

© 2024 Kohandel Gargari O et al. *JAMA Network Open.*

eFigure 18. Network Meta-Analysis Heatmap for Headache Duration

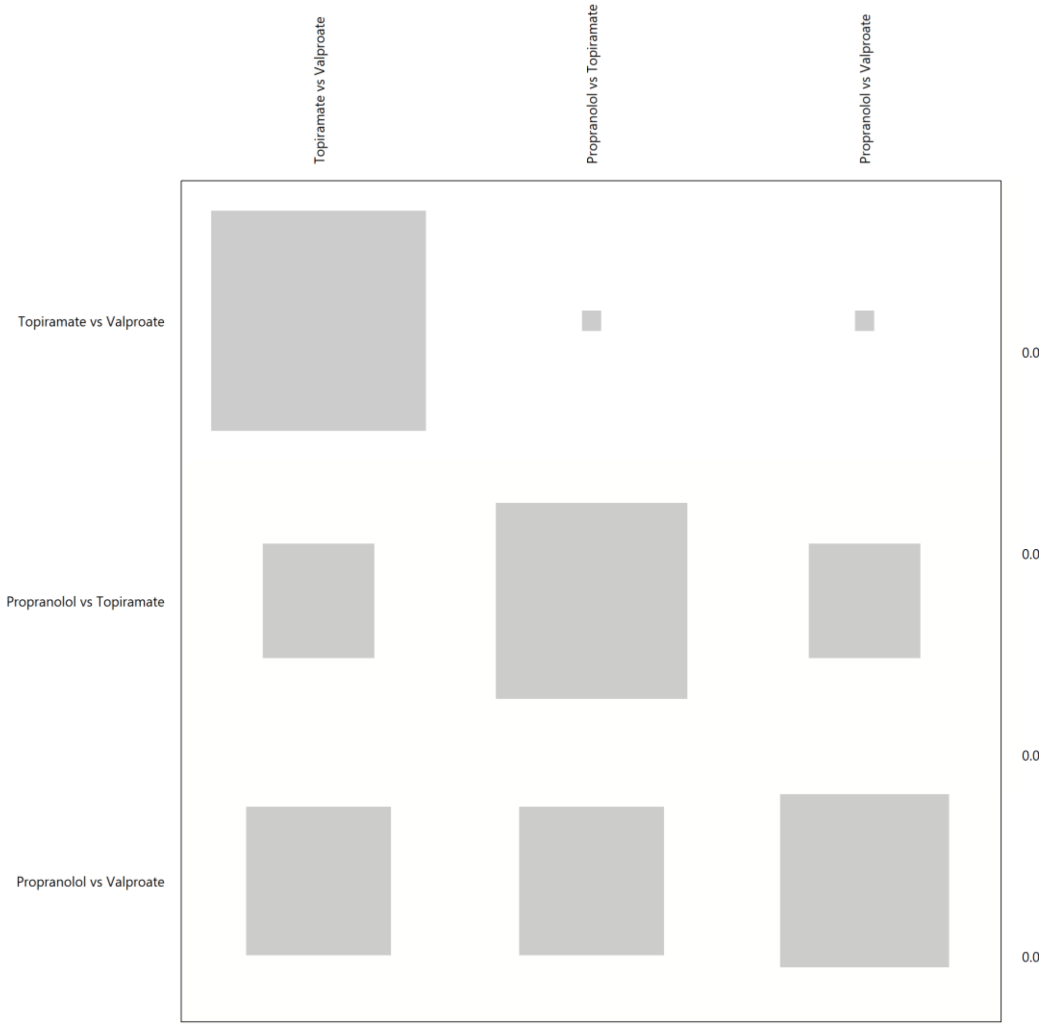

The net heat plot is a matrix visualization that highlights areas of inconsistency within the network meta-analysis. Each gray square's area represents the contribution of the direct estimate from the design in the column to the network estimate in the row. The colors indicate the change in inconsistency when relaxing the consistency assumption for single designs: cool colors (e.g., blue) indicate an increase in inconsistency, while warm colors (e.g., red) indicate a decrease. Diagonal colors show the inconsistency contribution of the corresponding design, whereas off-diagonal colors reflect the change in inconsistency between direct and indirect evidence. Clustering identifies hot spots of inconsistency, helping to locate potential sources for further investigation. Designs involving three or more treatments are marked with an underscore following the treatments of the design.

© 2024 Kohandel Gargari O et al. *JAMA Network Open.*

eFigure 19. Forest Plot of Adverse Events of Each Intervention Compared With Placebo

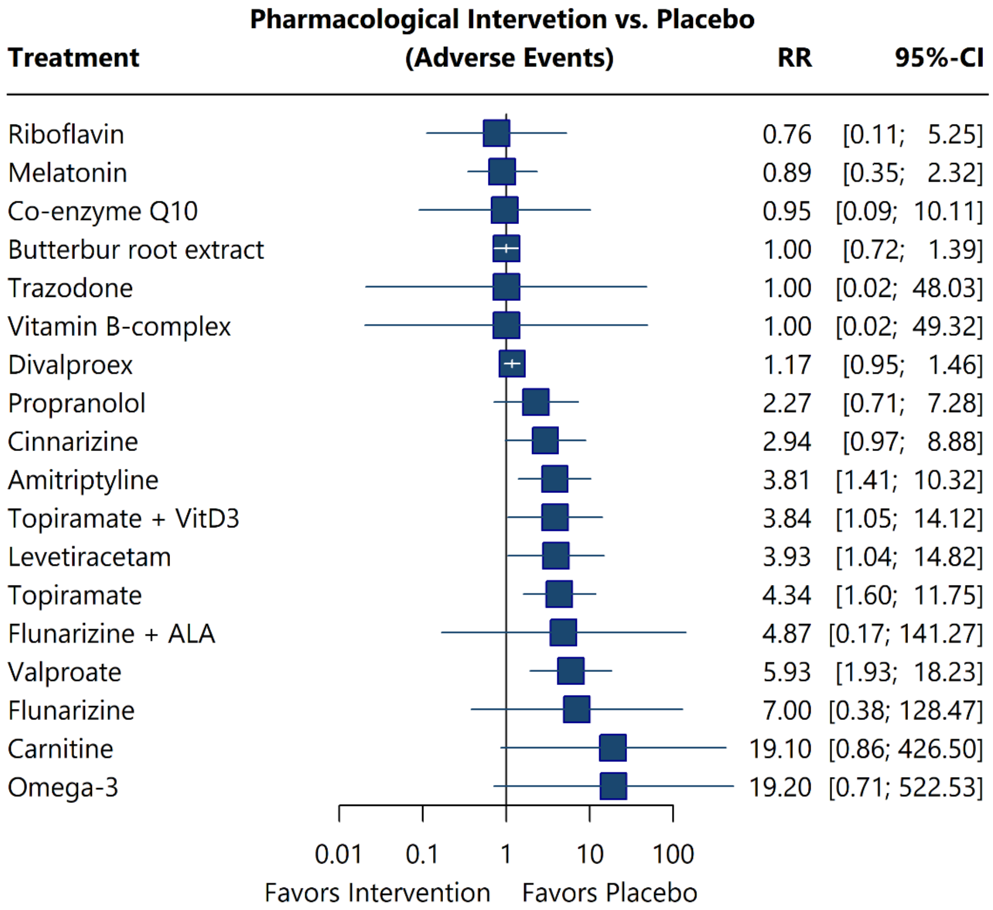

© 2024 Kohandel Gargari O et al. *JAMA Network Open.*

eFigure 20. Network Graph for Adverse Events

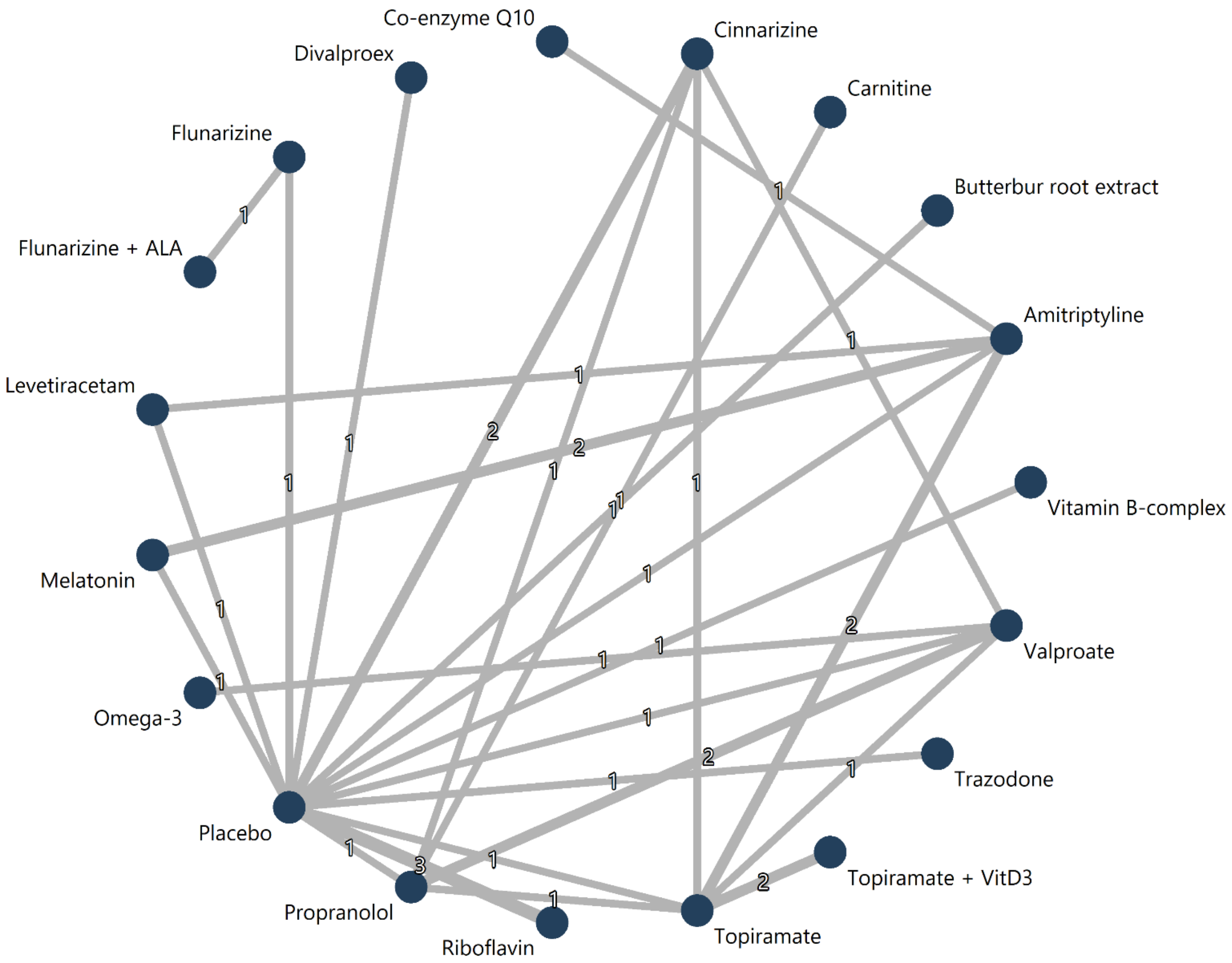

© 2024 Kohandel Gargari O et al. *JAMA Network Open.*

eFigure 21. Funnel Plot for Safety

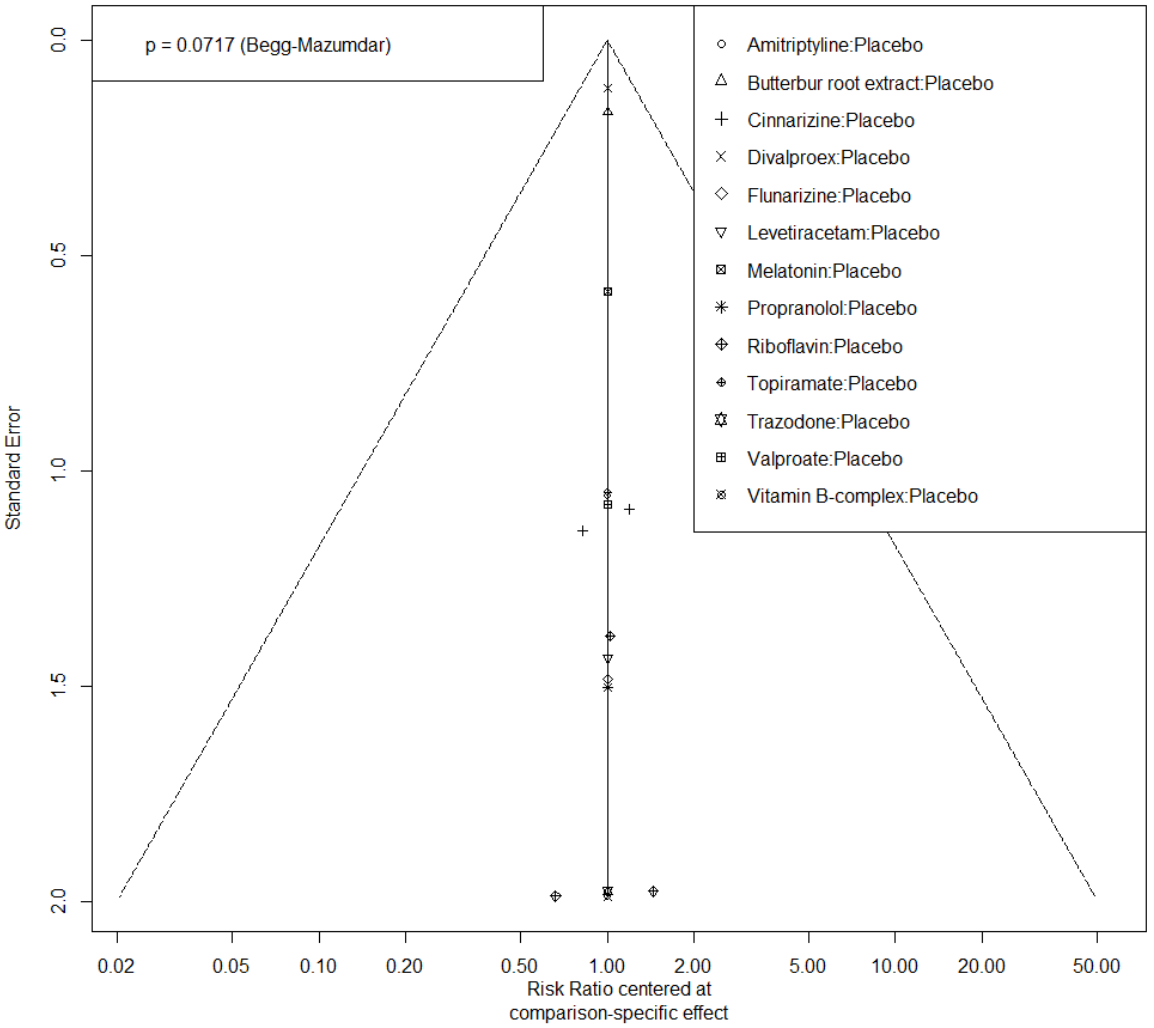

© 2024 Kohandel Gargari O et al. *JAMA Network Open.*

eTable 8. Network League Table for Adverse Events
This table presents the network league table for adverse events, comparing the relative safety of different interventions. The table displays the risk ratios (RR) for adverse events between each pair of interventions, with corresponding 95% confidence intervals (CIs).

1	Amitriptyline	.	.	.	4.00 (0.47; 34.07)	.	.	.	1.20 (0.41; 3.51)	2.72 (0.84; 8.78)	.	3.50 (0.44; 27.86)	.	.	0.98 (0.42; 2.30)	.	.	.	.
2	3.81 (1.33; 10.88)	Butterbur root extract	.	.	.	.	.	.	.	.	.	1.00 (0.72; 1.39)	.	.	.	.	.	.	.
3	0.20 (0.01; 4.32)	0.05 (0.00; 1.19)	Carnitine	.	.	.	.	.	.	.	.	.	8.43 (0.47; 149.89)	.	.	.	.	.	.
4	1.30 (0.44; 3.80)	0.34 (0.11; 1.08)	6.49 (0.30; 138.38)	Cinnarizine	.	.	.	.	.	.	.	3.57 (0.76; 16.70)	0.98 (0.02; 48.67)	.	0.57 (0.20; 1.65)	.	.	0.60 (0.15; 2.38)	.
5	4.00 (0.47; 34.07)	1.05 (0.10; 11.40)	20.05 (0.47; 850.69)	3.09 (0.28; 33.94)	Co-enzyme Q10	.	.	.	.	.	.	.	.	.	.	.	.	.	.
6	3.25 (1.17; 9.00)	0.85 (0.57; 1.26)	16.27 (0.72; 366.04)	2.51 (0.81; 7.72)	0.81 (0.08; 8.70)	Divalproex	.	.	.	.	.	1.17 (0.95; 1.46)	.	.	.	.	.	.	.
7	0.54 (0.03; 11.79)	0.14 (0.01; 2.67)	2.73 (0.04; 192.44)	0.42 (0.02; 9.44)	0.14 (0.00; 5.78)	0.17 (0.01; 3.10)	Flunarizine	1.44 (0.26; 7.83)	.	.	.	7.00 (0.38; 128.47)	.	.	.	.	.	.	.
8	0.78 (0.02; 26.22)	0.21 (0.01; 6.05)	3.92 (0.04; 382.95)	0.60 (0.02; 20.91)	0.20 (0.00; 11.97)	0.24 (0.01; 7.04)	1.44 (0.26; 7.83)	Flunarizine + ALA	.	.	.	.	.	.	.	.	.	.	.
9	0.97 (0.35; 2.66)	0.25 (0.06; 1.00)	4.86 (0.19; 121.80)	0.75 (0.18; 3.14)	0.24 (0.02; 2.59)	0.30 (0.08; 1.15)	1.78 (0.07; 43.62)	1.24 (0.03; 46.25)	Levetiracetam	.	.	17.00 (1.02; 283.18)	.	.	.	.	.	.	.
10	4.26 (1.62; 11.19)	1.12 (0.41; 3.06)	21.35 (0.91; 502.70)	3.29 (0.92; 11.71)	1.07 (0.10; 11.16)	1.31 (0.49; 3.48)	7.83 (0.37; 167.17)	5.44 (0.16; 180.22)	4.39 (1.14; 16.96)	Melatonin	.	0.58 (0.19; 1.83)	.	.	.	.	.	.	.
11	0.20 (0.01; 5.28)	0.05 (0.00; 1.44)	0.99 (0.01; 74.65)	0.15 (0.01; 3.93)	0.05 (0.00; 2.50)	0.06 (0.00; 1.68)	0.36 (0.00; 29.77)	0.25 (0.00; 28.38)	0.20 (0.01; 6.24)	0.05 (0.00; 1.34)	Omega-3	.	.	.	.	.	.	3.24 (0.14; 72.41)	.
12	3.81 (1.41; 10.32)	1.00 (0.72; 1.39)	19.10 (0.86; 426.50)	2.94 (0.97; 8.88)	0.95 (0.09; 10.11)	1.17 (0.95; 1.46)	7.00 (0.38; 128.47)	4.87 (0.17; 141.27)	3.93 (1.04; 14.82)	0.89 (0.35; 2.32)	19.20 (0.71; 522.53)	Placebo	0.17 (0.01; 3.32)	1.31 (0.19; 9.05)	0.25 (0.03; 1.93)	.	1.00 (0.02; 48.03)	0.20 (0.02; 1.69)	1.00 (0.02; 49.32)
13	1.68 (0.57; 4.97)	0.44 (0.13; 1.48)	8.43 (0.47; 149.89)	1.30 (0.46; 3.66)	0.42 (0.04; 4.64)	0.52 (0.16; 1.70)	3.09 (0.13; 71.03)	2.15 (0.06; 75.88)	1.73 (0.41; 7.37)	0.39 (0.11; 1.45)	8.47 (0.34; 211.82)	0.44 (0.14; 1.42)	Propranolol	.	0.56 (0.20; 1.54)	.	.	0.30 (0.10; 0.93)	.
14	5.00 (0.57; 43.90)	1.31 (0.19; 9.30)	25.08 (0.65; 971.38)	3.86 (0.42; 35.70)	1.25 (0.06; 26.42)	1.54 (0.22; 10.75)	9.19 (0.28; 301.84)	6.39 (0.13; 310.07)	5.16 (0.50; 53.70)	1.17 (0.14; 10.10)	25.21 (0.55; 1156.80)	1.31 (0.19; 9.05)	2.98 (0.31; 28.39)	Riboflavin	.	.	.	.	.
15	0.88 (0.40; 1.93)	0.23 (0.08; 0.66)	4.40 (0.22; 87.29)	0.68 (0.30; 1.54)	0.22 (0.02; 2.15)	0.27 (0.10; 0.75)	1.61 (0.07; 34.98)	1.12 (0.03; 37.64)	0.91 (0.26; 3.17)	0.21 (0.07; 0.63)	4.43 (0.18; 108.71)	0.23 (0.09; 0.62)	0.52 (0.24; 1.16)	0.18 (0.02; 1.54)	Topiramate	1.13 (0.49; 2.60)	.	0.79 (0.26; 2.40)	.
16	0.99 (0.31; 3.13)	0.26 (0.07; 1.00)	4.97 (0.22; 110.49)	0.77 (0.24; 2.47)	0.25 (0.02; 2.82)	0.31 (0.08; 1.14)	1.82 (0.08; 44.13)	1.27 (0.03; 46.86)	1.02 (0.23; 4.61)	0.23 (0.06; 0.94)	5.00 (0.18; 136.59)	0.26 (0.07; 0.96)	0.59 (0.19; 1.87)	0.20 (0.02; 2.03)	1.13 (0.49; 2.60)	Topiramate + VitD3	.	.	.
17	3.81 (0.07; 207.61)	1.00 (0.02; 48.70)	19.10 (0.13; 2733.44)	2.94 (0.05; 164.89)	0.95 (0.01; 88.86)	1.17 (0.02; 56.73)	7.00 (0.06; 888.25)	4.87 (0.03; 824.26)	3.93 (0.07; 235.52)	0.89 (0.02; 48.21)	19.20 (0.12; 3117.20)	1.00 (0.02; 48.03)	2.27 (0.04; 129.28)	0.76 (0.01; 57.63)	4.34 (0.08; 236.29)	3.84 (0.06; 228.34)	Trazodone	.	.
18	0.64 (0.22; 1.85)	0.17 (0.05; 0.54)	3.22 (0.16; 64.66)	0.50 (0.20; 1.26)	0.16 (0.01; 1.75)	0.20 (0.06; 0.62)	1.18 (0.05; 26.73)	0.82 (0.02; 28.61)	0.66 (0.16; 2.76)	0.15 (0.04; 0.54)	3.24 (0.14; 72.41)	0.17 (0.05; 0.52)	0.38 (0.16; 0.89)	0.13 (0.01; 1.20)	0.73 (0.34; 1.58)	0.65 (0.21; 2.02)	0.17 (0.00; 9.51)	Valproate	.
19	3.81 (0.07; 213.01)	1.00 (0.02; 50.01)	19.10 (0.13; 2790.61)	2.94 (0.05; 169.15)	0.95 (0.01; 90.90)	1.17 (0.02; 58.25)	7.00 (0.05; 907.29)	4.87 (0.03; 840.93)	3.93 (0.06; 241.50)	0.89 (0.02; 49.47)	19.20 (0.12; 3180.78)	1.00 (0.02; 49.32)	2.27 (0.04; 132.60)	0.76 (0.01; 59.01)	4.34 (0.08; 242.44)	3.84 (0.06; 234.15)	1.00 (0.00; 243.32)	5.93 (0.10; 342.55)	Vitamin B-complex

© 2024 Kohandel Gargari O et al. *JAMA Network Open.*

eFigure 22. Net Splitting Analysis for Adverse Events

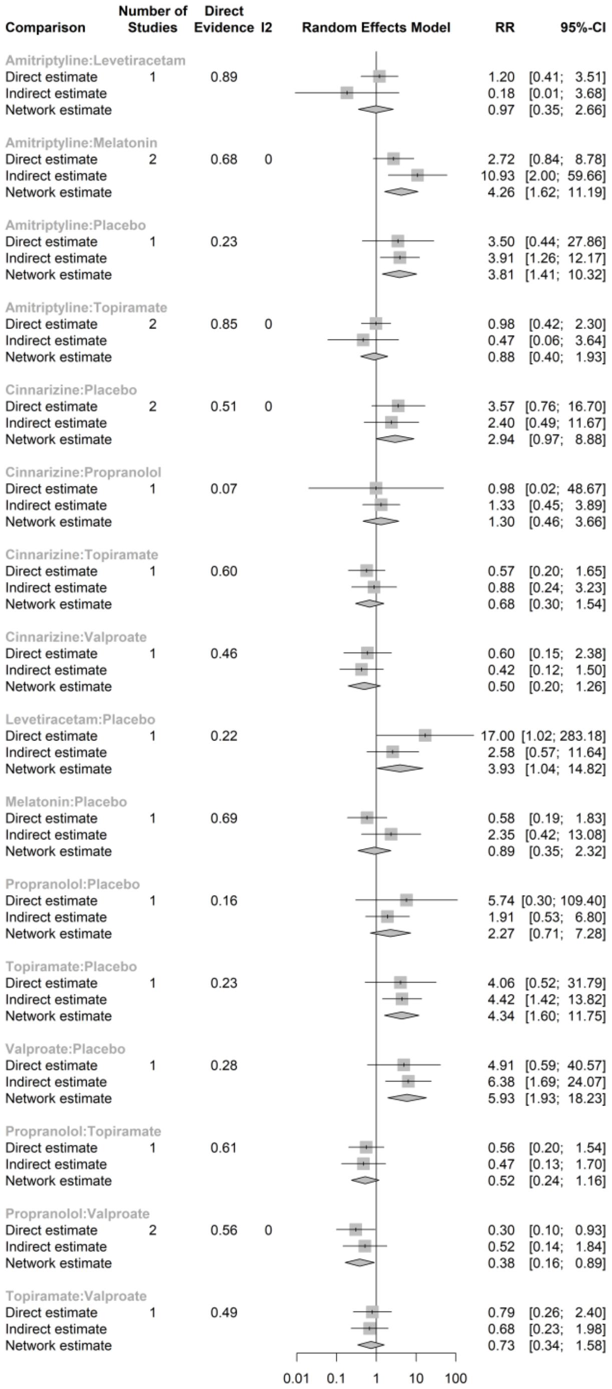

This figure presents the network splitting analysis for adverse events, which examines the consistency between direct and indirect evidence within the network meta-analysis. By splitting the network into these two types of evidence, the analysis evaluates whether the conclusions drawn from the meta-analysis are consistent regardless of the source of evidence, ensuring the robustness and reliability of the overall results.

© 2024 Kohandel Gargari O et al. *JAMA Network Open.*

eFigure 23. Network Meta-Analysis Heatmap for Adverse Events

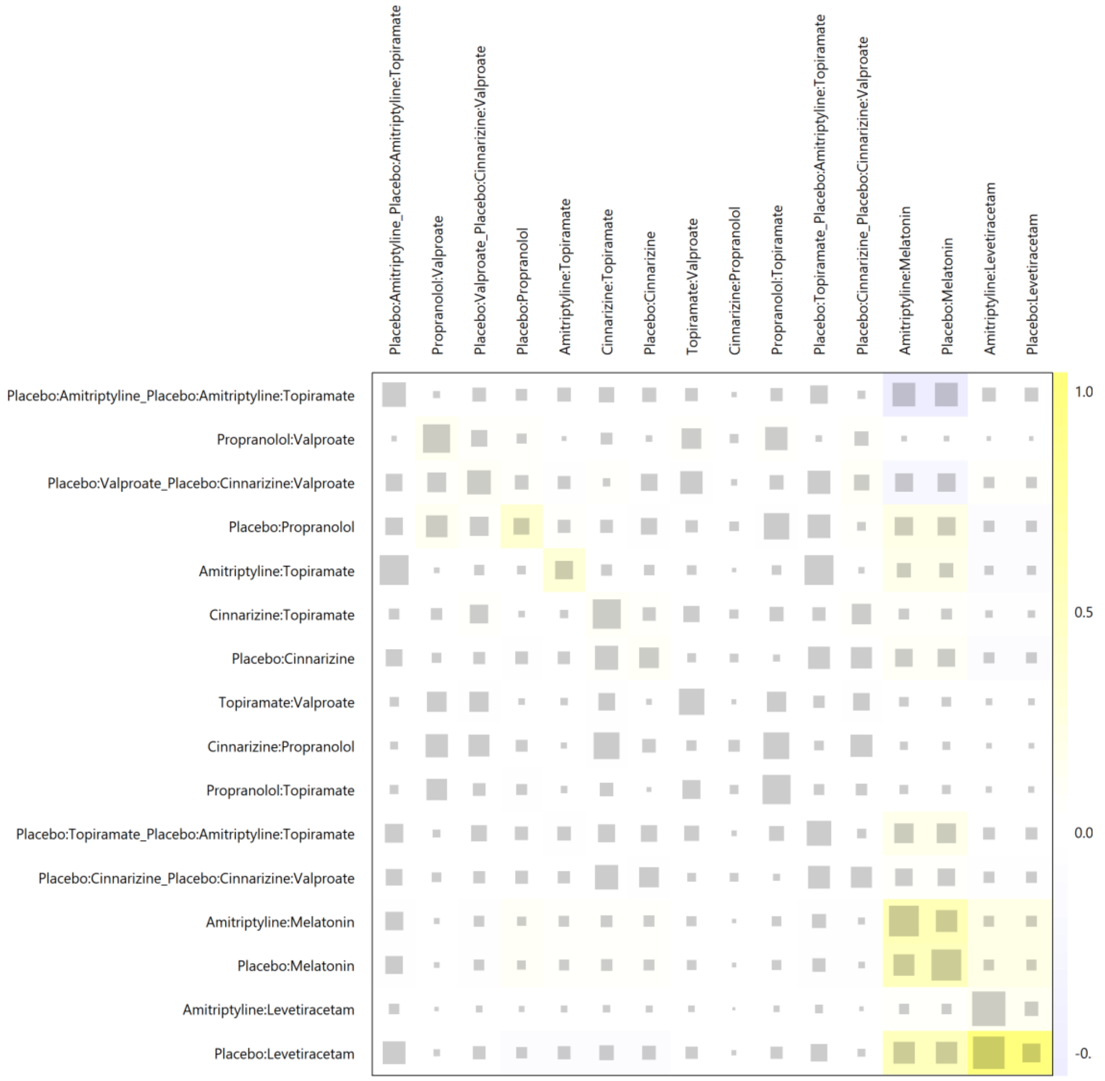

The net heat plot is a matrix visualization that highlights areas of inconsistency within the network meta-analysis. Each gray square's area represents the contribution of the direct estimate from the design in the column to the network estimate in the row. The colors indicate the change in inconsistency when relaxing the consistency assumption for single designs: cool colors (e.g., blue) indicate an increase in inconsistency, while warm colors (e.g., red) indicate a decrease. Diagonal colors show the inconsistency contribution of the corresponding design, whereas off-diagonal colors reflect the change in inconsistency between direct and indirect evidence. Clustering identifies hot spots of inconsistency, helping to locate potential sources for further investigation. Designs involving three or more treatments are marked with an underscore following the treatments of the design

© 2024 Kohandel Gargari O et al. *JAMA Network Open.*

eReferences.

1. Amanat M, Togha M, Agah E, Ramezani M, Tavasoli AR, Azizi Malamiri R, et al. Cinnarizine and sodium valproate as the preventive agents of pediatric migraine: A randomized double-blind placebo-controlled trial. Cephalalgia. 2020;40(7):665-74.

2. Amini L, Yaghini O, Ghazavi M, Aslani N. L-carnitine versus Propranolol for pediatric migraine prophylaxis. Iran J Child Neurol. 2021;15(2):77-86.

3. Apostol G, Cady RK, Laforet GA, Robieson WZ, Olson E, Abi-Saab WM, et al. Divalproex Extended-Release in Adolescent Migraine Prophylaxis: Results of a Randomized, Double-Blind, Placebo-Controlled Study. Headache: The Journal of Head and Face Pain. 2008;48(7):1012-25.

4. Ashrafi MR, Shabanian R, Zamani GR, Mahfelati F. Sodium Valproate versus Propranolol in paediatric migraine prophylaxis. European Journal of Paediatric Neurology. 2005;9(5):333-8.

5. Ashrafi MR, Najafi Z, Shafiei M, Heidari K, Togha M. Cinnarizine versus Topiramate in Prophylaxis of Migraines among Children and Adolescents: A Randomized, Double-Blind Clinical Trial. Iran J Child Neurol. 2014;8(4):18-27.

6. Ashrafi MR, Salehi S, Malamiri RA, Heidari M, Hosseini SA, Samiei M, et al. Efficacy and safety of cinnarizine in the prophylaxis of migraine in children: a double-blind placebo-controlled randomized trial. Pediatr Neurol. 2014;51(4):503-8.

7. Bakhshandeh Bali M, Rahbarimanesh AA, Sadeghi M, Sedighi M, Karimzadeh P, Ghofrani M. Comparison of propranolol and pregabalin for prophylaxis of childhood migraine: a randomised controlled trial. Acta Med Iran. 2015;53(5):276-80.

8. Battistella PA, Ruffilli R, Moro R, Fabiani M, Bertoli S, Antolini A, et al. A placebo-controlled crossover trial of nimodipine in pediatric migraine. Headache. 1990;30(5):264-8.

9. Battistella PA, Ruffilli R, Cernetti R, Pettenazzo A, Baldin L, Bertoli S, et al. A placebo-controlled crossover trial using trazodone in pediatric migraine. Headache. 1993;33(1):36-9.

10. Bidabadi E, Mashouf M. A Randomized Trial of Propranolol versus Sodium Valproate for the Prophylaxis of Migraine in Pediatric Patients. Pediatric Drugs. 2010;12(4):269-75.

11. Bruijn J, Duivenvoorden H, Passchier J, Locher H, Dijkstra N, Arts WF. Medium-dose riboflavin as a prophylactic agent in children with migraine: a preliminary placebo-controlled, randomised, double-blind, cross-over trial. Cephalalgia. 2010;30(12):1426-34.

12. Dalrymple RA, Wacogne I. Amitriptyline and topiramate are no better than placebo for childhood migraine. Arch Dis Child Educ Pract Ed. 2017;102(6):332.

© 2024 Kohandel Gargari O et al. *JAMA Network Open.*

13. Kotb Elmala M, Suliman HA, Al-Shokary AH, Ibrahim AO, Kamal NM, Elshorbagy HH, et al. The Impact of Vitamin D(3) Supplementation to Topiramate Therapy on Pediatric Migraine Prophylaxis. J Child Neurol. 2022;37(10-11):833-9.

14. Fallah R, Divanizadeh MS, Karimi M, Mirouliaei M, Shamszadeh A. Topiramate and Propranolol for Prophylaxis of Migraine. The Indian Journal of Pediatrics. 2013;80(11):920-4.

15. Fallah R, Fazelishoroki F, Sekhavat L. A Randomized Clinical Trial Comparing the Efficacy of Melatonin and Amitriptyline in Migraine Prophylaxis of Children. Iran J Child Neurol. 2018;12(1):47-54.

16. Fallah R, Sarraf Yazd S, Sohrevardi SM. Efficacy of Topiramate Alone and Topiramate Plus Vitamin D3 in the Prophylaxis of Pediatric Migraine: A Randomized Clinical Trial. Iran J Child Neurol. 2020;14(4):77-86.

17. Fayyazi A, Khajeh A, Ghazavi A, Sangestani M. Omega 3 in Childhood Migraines: a Double Blind Randomized Clinical Trial. Iran J Child Neurol. 2016;10(1):9-13.

18. Fayyazi A, Abdollahi A, Moradi A, Bazmamoun H. Administration in Efficacy of Melatonin Reducing Headaches in Children With Migraines and Sleep Disorders A Randomized Clinical Trial Study. Iranian Journal of Child Neurology. 2022;16(4):55-64.

19. Gelfand AA, Allen IE, Grimes B, Irwin S, Qubty W, Greene K, et al. Melatonin for migraine prevention in children and adolescents: A randomized, double-blind, placebo-controlled trial after single-blind placebo lead-in. Headache. 2023.

20. Ghazavi M, Jelodar G, Yaghini O, Nasiri J, Sadeghian S, Malamiri RA. The efficacy and safety of levetiracetam in the prophylaxis of migraine headaches in children - A randomised trial. Pediatria Polska. 2019;94(3):145-50.

21. Gibler RC, Peugh JL, Coffey CS, Chamberlin LA, Ecklund D, Klingner E, et al. Impact of preventive pill-based treatment on migraine days: A secondary outcome study of the Childhood and Adolescent Migraine Prevention (CHAMP) trial and a comparison of self-report to nosology-derived assessments. Headache. 2023;63(6):805-12.

22. Gillies D, Sills M, Forsythe I. Pizotifen (Sanomigran) in childhood migraine. A double-blind controlled trial. Eur Neurol. 1986;25(1):32-5.

23. Jafari N, Nasehi MM, Nasiri Eghbali A, Taghdiri MM, Karimzadeh P. Comparing Pregabalin and Sodium Valproate in Pediatric Migraine Prophylaxis: A Randomized Clinical Trial. Iran J Child Neurol. 2023;17(3):119-29.

24. Katibeh P, Shirazi RB, Shiraly R. Comparing the efficacy of propranolol+ cinarizine and propranolol+placebo in controlling pediatric migraine headaches: A randomized controlled trial. J Mazandaran Univ Med Sci. 2021;31(192):44-53.

25. Keerthana D, Mishra D, Chauhan MK, Juneja M. Effect of Propranolol Prophylaxis on Headache Frequency in Children with Migraine Without Aura: A Randomized, Double-Blind, Placebo-Controlled Trial. Indian J Pediatr. 2023;90(9):880-5.

© 2024 Kohandel Gargari O et al. *JAMA Network Open.*

26. Lakshmi CVS, Singhi P, Malhi P, Ray M. Topiramate in the Prophylaxis of Pediatric Migraine: A Double-Blind Placebo-Controlled Trial. Journal of Child Neurology. 2007;22(7):829-35.

27. Lewis D, Winner P, Saper J, Ness S, Polverejan E, Wang S, et al. Randomized, Double-Blind, Placebo-Controlled Study to Evaluate the Efficacy and Safety of Topiramate for Migraine Prevention in Pediatric Subjects 12 to 17 Years of Age. Pediatrics. 2009;123(3):924-34.

28. Ludvigsson J. PROPRANOLOL USED IN PROPHYLAXIS OF MIGRAINE IN CHILDREN. Acta Neurologica Scandinavica. 1974;50(1):109-15.

29. MacLennan, Suzanna C., Wade FM, Forrest KML, Ratanayake PD, Fagan E, Antony J. High-Dose Ribof lavin for Migraine Prophylaxis in Children: A Double-Blind, Randomized, Placebo-Controlled Trial. Journal of Child Neurology. 2008;23(11):1300-4.

30. Montazerlotfelahi H, Amanat M, Tavasoli AR, Agah E, Zamani GR, Sander JW, et al. Levetiracetam for prophylactic treatment of pediatric migraine: A randomized double-blind placebo-controlled trial. Cephalalgia. 2019;39(12):1509-17.

31. Oelkers-Ax R, Leins A, Parzer P, Hillecke T, Bolay HV, Fischer J, et al. Butterbur root extract and music therapy in the prevention of childhood migraine: An explorative study. European Journal of Pain. 2008;12(3):301-13.

32. Powers SW, Coffey CS, Chamberlin LA, Ecklund DJ, Klingner EA, Yankey JW, et al. Trial of Amitriptyline, Topiramate, and Placebo for Pediatric Migraine. N Engl J Med. 2017;376(2):115-24.

33. Puliappadamb HM, Satpathy AK, Mishra BR, Maiti R, Jena M. Evaluation of safety and efficacy of add-on Alpha-lipoic acid on migraine prophylaxis in an adolescent population: A randomized controlled trial. J Clin Pharmacol. 2023.

34. Sadeghvand S, Barzegar M, Shiva S, Tarmahi V, Khodaie H, Rahimi Khamaneh E, et al. The Effects of Vitamin B-Complex Supplementation on Serum Homocysteine Levels and Migraine Severity in Children A Randomized Controlled Trial. Iran J Child Neurol. 2023;17(3):141-53.

35. Santucci M, Cortelli P, Rossi PG, Baruzzi A, Sacquegna T. L-5-Hydroxytryptophan Versus Placebo in Childhood Migraine Prophylaxis: A Double-Blind Crossover Study. Cephalalgia. 1986;6(3):155-7.

36. Sezer T, Kandemir H, Alehan F. A randomized trial comparing amitriptyline versus topiramate for the prophylaxis of chronic daily headache in pediatric patients. Int J Neurosci. 2013;123(8):553-6.

37. Shahnawaz K, Mughal BB, Madni B, Malhi KA, Siddiqui MA. Comparison of efficacy and tolerability of melatonin and amitriptyline in children suffering with migraine. Med Forum Monthly. 2019;30(5):12-5.

38. Slater SK, Nelson TD, Kabbouche MA, LeCates SL, Horn P, Segers A, et al. A randomized, double-blinded, placebo-controlled, crossover, add-on study of CoEnzyme Q10 in the prevention of pediatric and adolescent migraine. Cephalalgia. 2011;31(8):897-905.

39. Sorge F, Marano E. Flunarizine v. placebo in childhood migraine. A double-blind study. Cephalalgia. 1985;5 Suppl 2:145-8.
© 2024 Kohandel Gargari O et al. *JAMA Network Open.*

40. Talebian A, Soltani B, Banafshe HR, Moosavi GA, Talebian M, Soltani S. Prophylactic effect of riboflavin on pediatric migraine: a randomized, double-blind, placebo-controlled trial. Electron Physician. 2018;10(2):6279-85.

41. Togha M, Malamiri RA, Rashidi-Ranjbar N, Asa S, Mahvelati F, Ashrafi MR. Efficacy and safety of cinnarizine in the prophylaxis of migraine headaches in children: an open, randomized comparative trial with propranolol. Acta Neurologica Belgica. 2012;112(1):51-5.

42. Tonekaboni SH, Ghazavi A, Fayyazi A, Khajeh A, Taghdiri MM, Gorji FA, et al. Prophylaxis of childhood migraine: Topiramate versus Propranolol. Iran J Child Neurol. 2013;7(1):9-14.

43. Winner P, Pearlman EM, Linder SL, Jordan DM, Fisher AC, Hulihan J, et al. Topiramate for Migraine Prevention in Children: A Randomized, Double-Blind, Placebo-Controlled Trial. Headache: The Journal of Head and Face Pain. 2005;45(10):1304-12.

44. Yadav R, Singh TP, Shukla SK. Sodium valproate versus topiramate for prophylaxis of migraine among children and adolescents: A randomised trial. J Indian Acad Clin Med. 2017;18(3):180-83.

45. Yaghini O, Hoseini N, Ghazavi MR, Mansouri V, Nasiri J, Moosavian T, et al. A Comparative Study on the Efficacy of Coenzyme Q10 and Amitriptyline in the Prophylactic Treatment of Migraine Headaches in Children: A Randomized Controlled Trial. Adv Biomed Res. 2022;11:43.

© 2024 Kohandel Gargari O et al. *JAMA Network Open.*
