# Supplementary material for: Preventive Medications in Pediatric Migraine: A Network Meta-Analysis
Source: JAMA Netw Open. 2024 Oct 10;7(10):e2438666. doi: 10.1001/jamanetworkopen.2024.38666 (PMC11581497; doi:10.1001/jamanetworkopen.2024.38666)
Supplement: Supplement 2. — Data Sharing Statement [file jamanetwopen-e2438666-s002.pdf]

Data Sharing Statement

Gargari. Effectiveness of Preventive Medications in Pediatric Migraine. *JAMA Netw Open*. Published October 10, 2024. doi:10.1001/jamanetworkopen.2024.38666

Data
Data available: No
